# AKR1D1 knockout mice develop a sex-dependent metabolic phenotype

**DOI:** 10.1530/JOE-21-0280

**Published:** 2022-03-23

**Authors:** Laura L Gathercole, Nikolaos Nikolaou, Shelley E Harris, Anastasia Arvaniti, Toryn M Poolman, Jonathan M Hazlehurst, Denise V Kratschmar, Marijana Todorčević, Ahmad Moolla, Niall Dempster, Ryan C Pink, Michael F Saikali, Liz Bentley, Trevor M Penning, Claes Ohlsson, Carolyn L Cummins, Matti Poutanen, Alex Odermatt, Roger D Cox, Jeremy W Tomlinson

**Affiliations:** 1 Oxford Centre for Diabetes, Endocrinology and Metabolism, NIHR Oxford Biomedical Research Centre, University of Oxford, Churchill Hospital, Oxford, UK; 2 Department of Biological and Medical Sciences, Oxford Brookes University, Oxford, UK; 3 Institute of Metabolism and Systems Research, University of Birmingham, Birmingham, UK; 4 Swiss Centre for Applied Human Toxicology and Division of Molecular and Systems Toxicology, Department of Pharmaceutical Sciences, University of Basel, Basel, Switzerland; 5 Department of Pharmaceutical Sciences, Leslie Dan Faculty of Pharmacy, University of Toronto, Toronto, Ontario, Canada; 6 Mammalian Genetics Unit, Medical Research Council Harwell, Oxford, UK; 7 Center of Excellence in Environmental Toxicology, Department of Systems Pharmacology & Translational Therapeutics, University of Pennsylvania Perelman School of Medicine, Philadelphia, Pennsylvania, USA; 8 Department of Internal Medicine and Clinical Nutrition, Institute of Medicine, The Sahlgrenska Academy, University of Gothenburg, Gothenburg, Sweden; 9 Institute of Biomedicine, Research Centre for Integrative Physiology and Pharmacology, University of Turku, Turku, Finland

**Keywords:** steroid, bile acid, cortisol, cholic acid, chenodeoxycholic acid, non-alcoholic fatty liver disease, metabolic syndrome

## Abstract

Steroid 5β-reductase (AKR1D1) plays important role in hepatic bile acid synthesis and glucocorticoid clearance. Bile acids and glucocorticoids are potent metabolic regulators, but whether AKR1D1 controls metabolic phenotype *in vivo* is unknown. *Akr1d1^–/–^* mice were generated on a C57BL/6 background. Liquid chromatography/mass spectrometry, metabolomic and transcriptomic approaches were used to determine effects on glucocorticoid and bile acid homeostasis. Metabolic phenotypes including body weight and composition, lipid homeostasis, glucose tolerance and insulin tolerance were evaluated. Molecular changes were assessed by RNA-Seq and Western blotting. Male *Akr1d1^–/–^* mice were challenged with a high fat diet (60% kcal from fat) for 20 weeks. *Akr1d1^–/–^* mice had a sex-specific metabolic phenotype. At 30 weeks of age, male, but not female, *Akr1d1^–/–^* mice were more insulin tolerant and had reduced lipid accumulation in the liver and adipose tissue yet had hypertriglyceridemia and increased intramuscular triacylglycerol. This phenotype was associated with sexually dimorphic changes in bile acid metabolism and composition but without overt effects on circulating glucocorticoid levels or glucocorticoid-regulated gene expression in the liver. Male *Akr1d1^–/–^* mice were not protected against diet-induced obesity and insulin resistance. In conclusion, this study shows that AKR1D1 controls bile acid homeostasis *in vivo* and that altering its activity can affect insulin tolerance and lipid homeostasis in a sex-dependent manner.

## Introduction

Bile acids and steroid hormones (including glucocorticoids) are potent regulators of metabolism and energy balance. Bile acid sequestrants improve metabolic phenotype (Kobayashi *et al.* 2007), and patients with glucocorticoid excess, Cushing’s syndrome, develop broad adverse metabolic features (Newell-Price *et al.* 2006).

The metabolic effects of bile acids are primarily mediated through the farnesoid X receptor (FXR) and Takeda G-protein receptor 5 (TGR5); however, bile acids as well as intermediates of their synthesis can also activate or antagonise multiple metabolic receptors, including the liver X receptor and pregnane X receptor (PXR) (Chiang 2002). The primary bile acids, cholic acid (CA), chenodeoxycholic acid (CDCA), and in mice α- and β-murocholic acid (α/β-MCA), are synthesised from cholesterol in the liver and, once released into the intestine, can be further metabolised by bacterial enzymes to secondary bile acids, deoxycholic acid (DCA), lithocholic acid (LCA) and ω-MCA (Vaz & Ferdinandusse 2017). As bile acid receptors have differing affinities for each bile acid species, metabolic consequences are dependent on both total bile acid levels and composition of the bile acid pool. This is highlighted by the phenotype of *Cyp8b1^–/–^* mice, which lack the sterol 12α-hydroxylase required for the generation of CA. These animals have a complete absence of CA and its derivatives, and metabolically, they are more insulin sensitive and protected against diet-induced obesity (Kaur *et al.* 2015, Bertaggia *et al.* 2017).

Glucocorticoid’s availability to bind its receptor is not only dependent on circulating levels but also on the tissue-specific complement of pre-receptor steroid metabolising enzymes. Best described are 11β-hydroxysteroid dehydrogenase 1 (11β-HSD1) and the 5α-reductases type 1 and 2 (5αR1 and 2). 11β-HSD1 converts the inactive glucocorticoid cortisone to its active form cortisol, and *11β-Hsd1^–/–^* mice have a beneficial metabolic phenotype with improved insulin sensitivity and protection against hepatic steatosis (Morton *et al.* 2001). 5αRs catalyse the first step in cortisol clearance towards 5α-tetrahydrocortisol formation, and 5αR1*^–/–^* mice have increased hepatic steatosis on a Western diet (Dowman *et al.* 2013, Livingstone *et al.* 2015), whilst patients treated with 5αR inhibitors have increased intrahepatic lipid accumulation (Hazlehurst *et al.* 2016), skeletal muscle insulin resistance (Upreti *et al.* 2014) and risk of type 2 diabetes (Wei *et al.* 2019).

The enzyme ∆4-3-oxosteroid 5β-reductase is encoded by the gene *AKR1D1* (named *Akr1d1* or *Akr1d4* in mice) and catalyses an essential step in bile acid synthesis, with 5β-reduction being required for the generation of both CA and CDCA (Chen & Penning 2014). AKR1D1 has also the 5β-reductase for all C19-C27 steroids (which include glucocorticoids and bile acids). It plays an important role in glucocorticoid clearance where, analogous to 5αR, AKR1D1 is the first step in the clearance of cortisol to 5β-tetrahydrocortisol and cortisone to 5β-tetrahydrocortisone. Patients with loss of function mutations in AKR1D1 have altered glucocorticoid and bile acid metabolism (Palermo *et al.* 2008); urinary bile acids are almost absent, suggesting a more pronounced effect on bile acid homeostasis. These patients develop neonatal cholestasis thought to be due to an accumulation of toxic bile acid precursors and 5α-reduced (allo) bile acids, although there is evidence of spontaneous recovery. Nothing is known about their metabolic status (Palermo *et al.* 2008).

Despite being potentially central in the regulation of glucocorticoid and bile acid availability, the role of AKR1D1 in metabolic homeostasis is almost entirely unexplored (Nikolaou *et al.* 2021). We have recently shown that manipulating AKR1D1 activity *in vitro* alters glucocorticoid and bile acid action with effects on insulin signalling, as well as carbohydrate and lipid metabolism (Nikolaou *et al.* 2019*a*, *b*, 2020). To investigate its role in the regulation of metabolism *in vivo,* we generated an *Akr1d1^–/–^* mouse.

## Materials and methods

### Strain generation

The *Akr1d1*^–/–^ strain was generated from targeted embryonic stem (ES) cells obtained from the KOMP repository (www.komp.org; project ID VG12494). Mice were rederived to the Medical Research Council (MRC) Harwell Mary Lyon Centre-specified pathogen-free facility and maintained on C57BL/6NTac. The *Akr1d1* tm1 allele (*Akr1d1*^tm1(KOMP)Vlcg^) was converted to tm1.1 by cre recombination to remove the neo cassette. *Akr1d1*^tm1/+^mice were crossed to mice carrying a ubiquitously expressed cre (C57BL/6NTac-Tg(ACTB-cre)3Mrt/H). Offspring from this cross carrying the converted allele *Akr1d1*^tm1.1/+^, and the cre recombinase, were bred to C57BL/6NTac to remove the cre allele and *Akr1d1*^tm1.1/+^ were crossed to C57BL/6NTac to increase numbers. *Akr1d1*^tm1.1^heterozygotes were intercrossed to produce *Akr1d1*^tm1.1/tm1.1^ and *Akr1d1*^+/+^ littermates for phenotyping. *Akr1d1*^–/–^ showed normal Mendelian inheritance (385 mice: WT 102; Het 179: *Akr1d1*^–/–^104) and sex ratios (385 mice: female 189; male 196).

### Husbandry and experimental design

*Akr1d1^–/–^* mice were kept and studied in accordance with UK Home Office legislation and local ethical guidelines issued by the MRC (Responsibility in the Use of Animals for Medical Research, July 1993; home office license 30/3146). All procedures were conducted in accordance with the Animals (Scientific Procedures) Act 1986 Amendment Regulations 2012 (SI 4 2012/3039) and approved by the local Animal Welfare and Ethical Review Board. Mice were kept under controlled light (12  h light:12  h darkness cycle), temperature (21 ± 2°C) and humidity (55 ± 10%). They had free access to water (9–13 ppm chlorine) and standard diet (SDS Rat and Mouse No. 3 Breeding diet, RM3) until 10 weeks of age when they were transferred to a high fat (60% kcal from fat; D12492; Research Diets) or matched control diet (10% kcal from fat; D12450J; Research Diets).

Male and female cohorts were bred for longitudinal metabolic phenotyping. They were housed in single sex groups of mixed genotypes across multiple litters and were not randomised into groups. Experimental groups of 15 were used, with sample size estimates based on previous experience with mouse models in which metabolic traits were measured (Paterson *et al.* 2004, Dowman *et al.* 2013).

### Metabolic assessments

Body weight was measured weekly in the morning using average weights (g) calculated by Adventure Pro balances (OHAUS Europe GmbH, Nanikon, Switzerland). Fat and lean mass was assessed by Echo-MRI (Echo Medical System, Houston, Texas, USA) at 10 weeks of age and body composition by high-energy X-rays using the Lunar PIXImus (GE Healthcare, Chicago, USA) at 29 weeks.

Calorimetry data were collected in a PhenoMaster system (TSE Systems, Berlin, Germany) at 11 weeks of age. Data were collected at three to four time points each hour, and measurements included photobeam activity monitoring, food intake and indirect gas calorimetry that simultaneously measures oxygen consumption (VO_2_), carbon dioxide production (VCO_2_) and respiratory exchange ratio (RER). Fecal pellets from *n*  = 7 mice were collected over 24 h and stored at −20^o^C before energy content was measured by bomb calorimetry (IKA C2000 Basic, IKA Oxford, Oxford, UK) as previously described (Moir *et al.* 2016) and triacylglycerol by colorimetric assay (Cayman Chemical).

To measure intraperitoneal or oral glucose tolerance (ipGTT and oGTT), *n*  = 14–15 mice were fasted overnight, then either injected intraperitoneally with 20% glucose solution (2 g glucose/kg body weight; Sigma) or orally gavaged with 1 g glucose/kg body weight. Glucose concentration was measured in the tail vein blood of restrained animals at t = 0, 15, 30, 60 and 120 min (Alphatrak, Abbott). To measure intraperitoneal insulin tolerance (ipITT), mice were fasted for 4–5 h, then injected intraperitoneally with insulin at 0.75 IU/kg for females and 1.25 IU/kg for males (Hypurin Bovine Insulin). Glucose concentration was measured in the tail vein blood of restrained animals at t = 0, 15, 30, 45, 60 and 90 min (Alphatrak, Abbott). To minimise stress, animals were restraint acclimated before glucose tolerance and ipITT procedures.

### Blood biochemistry and metabolomics

At termination, mice were anaesthetised with isoflurane and blood was collected via retro-orbital bleed. Samples were kept on ice, then centrifuged for 10 min at 8000 ***g*** at room temperature. Corticosterone (Enzo Corticosterone ELISA Kit, Lausen, Switzerland), insulin (CrystalChem Ultra-Sensitive Mouse Insulin ELISA, Zaandam, Netherlands) and GLP-1 (CrystalChem Mouse GLP-1 ELISA, Zaandam, Netherlands) were measured by ELISA. Triacylglycerol, total cholesterol, LDL cholesterol, alanine transaminase and aspartate transaminase were measured using Instrumentation Laboratory kits on an ILab 650 Clinical Chemistry analyser with manufacturer-recommended reagents and settings. Unbiased plasma metabolomics was performed by Metabolon (Metabolon, Inc., Research Triangle Park, NC, USA) using their global mouse metabolite panel and according to published methods (Lawton *et al.* 2008). Metabolon data are presented as log2(FC) with *P* values generated from relative signal intensity from *n*  = 10 mice.

### LC-MS/MS quantification of bile acids and their intermediates and GC-MS quantification of sex steroids

Extraction and quantification of bile acids from plasma (25 µL) and liver tissue (30 ± 10 mg) was performed using the protocol from Penno *et al.* (2013) with the following modifications: Plasma samples were diluted with water (75 µL) and subjected to protein precipitation by isopropanol (900 µL, containing 100 nM internal standards). Samples were incubated at 4°C for 30 min and centrifuged at 4°C and 16,000 ***g*** for 10 min. Liver was homogenised at 4°C (three cycles: 30 s at 6500 rpm and 30 s break) in water–chloroform–methanol (1 mL; 20/20/60, v/v/v containing 100 nM internal standards) on a Precellys homogeniser (Bertin Instruments, Rockville, MD, USA) and incubated with continuous shaking at 37°C and 850 rpm for a further 15 min. Samples were centrifuged at room temperature and 16,000 ***g*** for 10 min, and 800 µL of supernatant was collected. Sample extractions were repeated twice. Injection volume was 2 µL for plasma and 3 µL for liver. Quantification was conducted as described in Penno *et al.* (2013) with minor modifications: eluent gradients were set from 0 to 8 min (25%), 8 to 17.5 min (35–68%), followed by a wash out 17.5 to 18 min (68–25%), 18 to 20 min (25–100%) and 20 to 22 min (100%). Flow rate was set to 0.63 mL/min. Experimental group sizes were *n*  = 11–15 mice. Statistical analysis of relative abundance was calculated from % of total bile acids using two-tailed unpaired parametric *t*-tests, and significance was defined by a false discovery rate (Benjamini, Krieger and Yekutieli method) adjusted *P* value < 1%. Principal component analysis was performed using the FactoMineR package (Lê *et al.* 2008) and the factoextra package to visualise the results in R.

Free and esterified oxysterols were measured as previously described (Magomedova & Cummins 2019) with the following modifications: Liver (100 mg) was spiked with 30 µL of 1 μM internal standard mix 25 (R/S), 26-hydroxycholesterol-d4, 7α-hydroxy-4-cholesten-3-one-d7, 7α,12α-dihydroxycholest-4-en-3-one-d7) (Toronto Research Chemicals, Ontario, Canada) and homogenised in chloroform/methanol (4 mL: CHCl_3_/MeOH, 2:1, v/v) containing 50 μg/mL butylated hydroxytoluene. Oxysterols were subsequently extracted by solid phase extraction using 100 mg Silica SPE columns (Waters, Hertfordshire, UK). Samples were dried under constant stream of N_2_ and reconstituted in 125 μL of methanol for analysis by LC-MS/MS. The transitions monitored were previously reported (Magomedova & Cummins 2019) with the addition of 7α-hydroxy-4-cholesten-3-one (401.3→383.0 m/z), 7α-hydroxy-4-cholesten-3-one-d7 (408.3→390.3 m/z), 7α,12α-dihydroxycholest-4-en-3-one (417.3→381.3 m/z) and 7α,12α-dihydroxycholest-4-en-3-one-d7 (424.3→388.3 m/z). Oxysterols were quantified relative to a calibration series ranging from 0.01 to 2 μM, and concentrations were calculated relative to their deuterated internal standards. Experimental group sizes were *n*  = 10 mice.

The concentrations of serum testosterone and 5α-dihydrotestosterone were determined with a validated gas chromatography tandem mass spectrometry method (Nilsson *et al.* 2015).

### Tissue histology and biochemistry

Adipose (gonadal and subcutaneous) and liver tissue was fixed in 4% buffered paraformaldehyde, samples were paraffin-embedded, and 5 μm sections were prepared on a microtome (Leica). Adipocyte area was determined as previously described (Small *et al.* 2018). Statistical significance was assessed using a Wilcoxon signed-rank test. Tissue triacylglycerol was measured in snap frozen tissue using a colorimetric assay (Cayman Chemical).

### RNA sequencing

Total liver RNA was extracted from *n*  = 10 mice using a RNeasy Plus mini kit (Qiagen). Total gonadal adipose RNA was extracted from *n*  = 10 mice using a modified Tri-reagent (Sigma-Aldrich) protocol. Tissue (50 mg) was homogenised in 1 mL of Tri-Reagent and incubated at room temperature for 5 min, 200 μL of chloroform was added, the sample was vigorously shaken and incubated at room temperature for 5 min. After centrifugation at 12,000 ***g*** for 15 min, the aqueous phase was combined with an equal volume of 70% ethanol, vortexed and transferred to an RNeasy Lipid Tissue spin column (Qiagen) for washing and elution. Concentration was determined spectrophotometrically at OD260 on a Nanodrop spectrophotometer (Thermo Scientific) and quality on an RNA Bioanalyzer chip (Agilent).

Following extraction, RNAs were incubated with oligo (dT) beads and enriched poly-A libraries were selected using TruSeq Stranded mRNA HT Sample Prep Kit for Illumina with custom 12 bp indexes. Libraries were multiplexed (10 samples per lane), clustered using HiSeq 3000/4000 PE Cluster Kit and paired-end sequenced (75 bp) using in-house indexes to a total depth of ~25 million read pairs, on the Illumina HiSeq4000 platform. Reads were mapped with Stampy (Lunter & Goodson 2011) on default settings with GRCm38/mm10 as genome reference and bam files merged using Rsamtools (v2.0). Gene-level read counts for all protein-coding RNA transcripts present in refTene mm10 were quantified in a strand-specific manner using FeatureCounts from the Rsubread package (v1.34.6). Differential expression analysis was performed using EdgeR (v3.26.6) (Robinson *et al.* 2010) on normalised genes counts using the trimmed mean of M-values (TMM) method for all autosomal protein-coding genes that were expressed at >0.25 counts per million in at least two samples. Statistical comparisons were performed using the glmLRT function in EdgeR and using an adjusted *P* value < 0.05% (Benjamini-Hochberg method). A full list of gene fold changes can be found in Supplementary datasets 1–4 (see section on supplementary materials given at the end of this article). Ingenuity pathway analysis (IPA, QIAGEN Redwood City, www.qiagen.com/ingeniuty) was used to predict causal networks and upstream regulators. The expression levels of key regulated genes were confirmed by quantitative PCR (qPCR) (Supplementary Table 2).

### RT and qPCR

Total RNA was extracted from snap frozen tissue (*n* = 10 liver) using Tri-Reagent (Sigma-Aldrich), and concentration was determined spectrophotometrically at OD260 on a Nanodrop spectrophotometer (Thermo Scientific). RT and qPCR were performed as previously described (Nikolaou *et al.* 2019*b*). The Ct of each sample was calculated using the following equation (where *E* is reaction efficiency determined from a standard curve): ΔCt = *E*^[min Ct-sample Ct]^ using the 1/40 dilution from a standard curve generated from a pool of all cDNAs as the calibrator. Relative expression ratio was calculated using the equation: ratio = ΔCt_[target]_/ΔCt_[ref],_and expression was normalised to the geometric mean of 18S rRNA and HPRT. Statistical analysis was performed on mean relative expression ratio values (ratio = ΔCt[target]/ΔCt).

### Statistics

Data are presented as mean ± s.d. unless otherwise stated. Data analysis was performed using Graphpad Prism software (Graphpad Software Inc). Normality was assessed using the Shapiro–Wilk test. Two-tailed, unpaired *t*-tests were used to compare differences in mean between genotype when assumptions of normal distribution were met with Mann–Whitney tests used on data sets with nonparametric distribution. Two-way ANOVA with Sidak corrections was used to compare means grouped by sex and genotype and repeated-measure two-way ANOVA for data collected across time. Comparisons were considered statistically significant at *P*  < 0.05.

## Results

### *Akr1d1* deletion decreases total bile acid levels and alters bile acid composition but does not affect glucocorticoid metabolism

**Figure 1:**
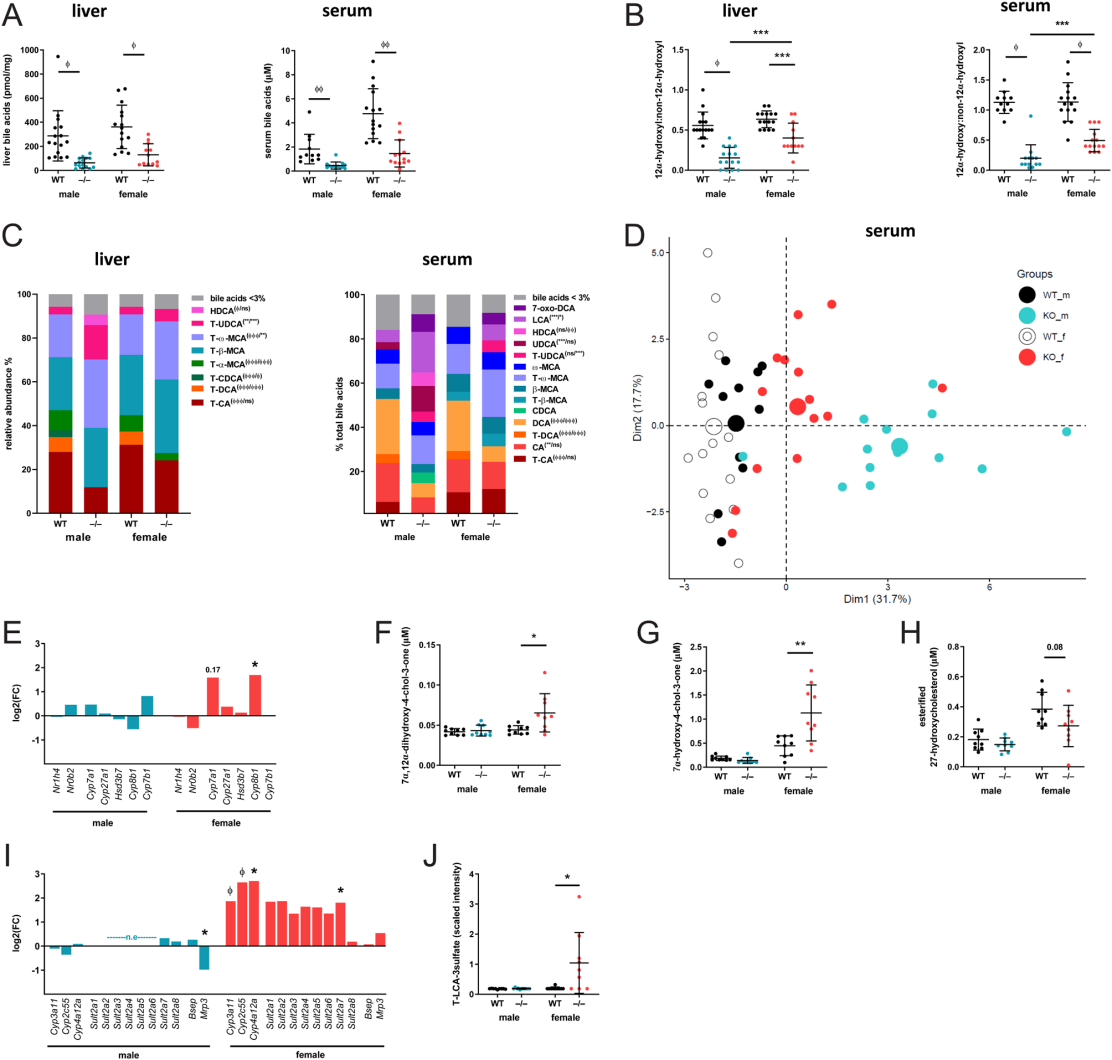
Hepatic and serum bile acids are lower in *Akr1d1^–/–^* mice with sexually dimorphic changes to bile acid metabolism and composition. Mature (30 weeks) male and female *Akr1d1^–/–^* mice have decreased total hepatic and serum bile acids (A) and altered bile acid composition with reduced 12α-hydroxylated/non-12α-hydroxylated bile ratio in the liver and serum (B and C) (*n*  = 12–16 mice). Principal component analysis shows greater divergence from WT in male *Akr1d1^–/–^* mice (D). *Akr1d1* deletion has a sexually dimorphic effect on mRNA expression of hepatic bile acid-metabolising genes and levels of bile acid intermediates. *Cyp8b1* expression is increased in *Akr1d1^–/–^* females but not in males (E) (*n*  = 10 mice) as are the AKR1D1 substrates 7α,12α-dihydroxy-4-chol-3-one (F) and 7α-hydroxy-4-chol-3-one (G) (*n*  = 10 mice). The oxysterol 27-hydroxycholesterol (27-OHC) is decreased in *Akr1d1^–/–^* females (H) (*n*  = 9 mice). Female *Akr1d1^–/–^* mice also have increased expression of the bile acid-detoxifying genes *Cyp3a11*, *Cyp2c55*, *Cyp4a12a* and *Sult2a7* (I) (*n*  = 10 mice) and serum levels of LCA sulphate (J) (*n*  = 9 mice). Data are presented as mean ± s.d., log2(FC), ratio or mean relative abundance. *P  < 0.05, **P  < 0.01, ***P  < 0.005, ^∅^ *P*  < 0.001, ^∅∅^ *P*  < 0.0005, ^∅∅∅^ *P*  < 0.0001 compared to WT. *P* values for bile acid composition compare WT and *Akr1d1^–/–^* within sex (male/female). (WT = WT C57BL/6; *–/–* = *Akr1d1^–/–^*). A full colour version of this figure is available at https://doi.org/10.1530/JOE-21-0280.

*Akr1d1* deletion (Supplementary Fig. 1A) had a marked impact on bile acid homeostasis. Total liver and serum (Fig. 1A) bile acid levels were reduced. In addition, composition was altered, with a decreased 12α-hydroxylated (CA and CA-derived)/non-12α-hydroxylated (CDCA and CDCA-derived) ratio in both the liver and serum (Fig. 1B and C). The relative reduction in 12α-hydroxylated bile acids was greater (Fig. 1B), and serum bile acid profiles were more markedly different (Fig. 1C and D) in male *Akr1d1^–/–^* mice. Absolute levels of liver and serum bile acids and bile acid intermediates are presented in Supplementary Table 1.

**Figure 2:**
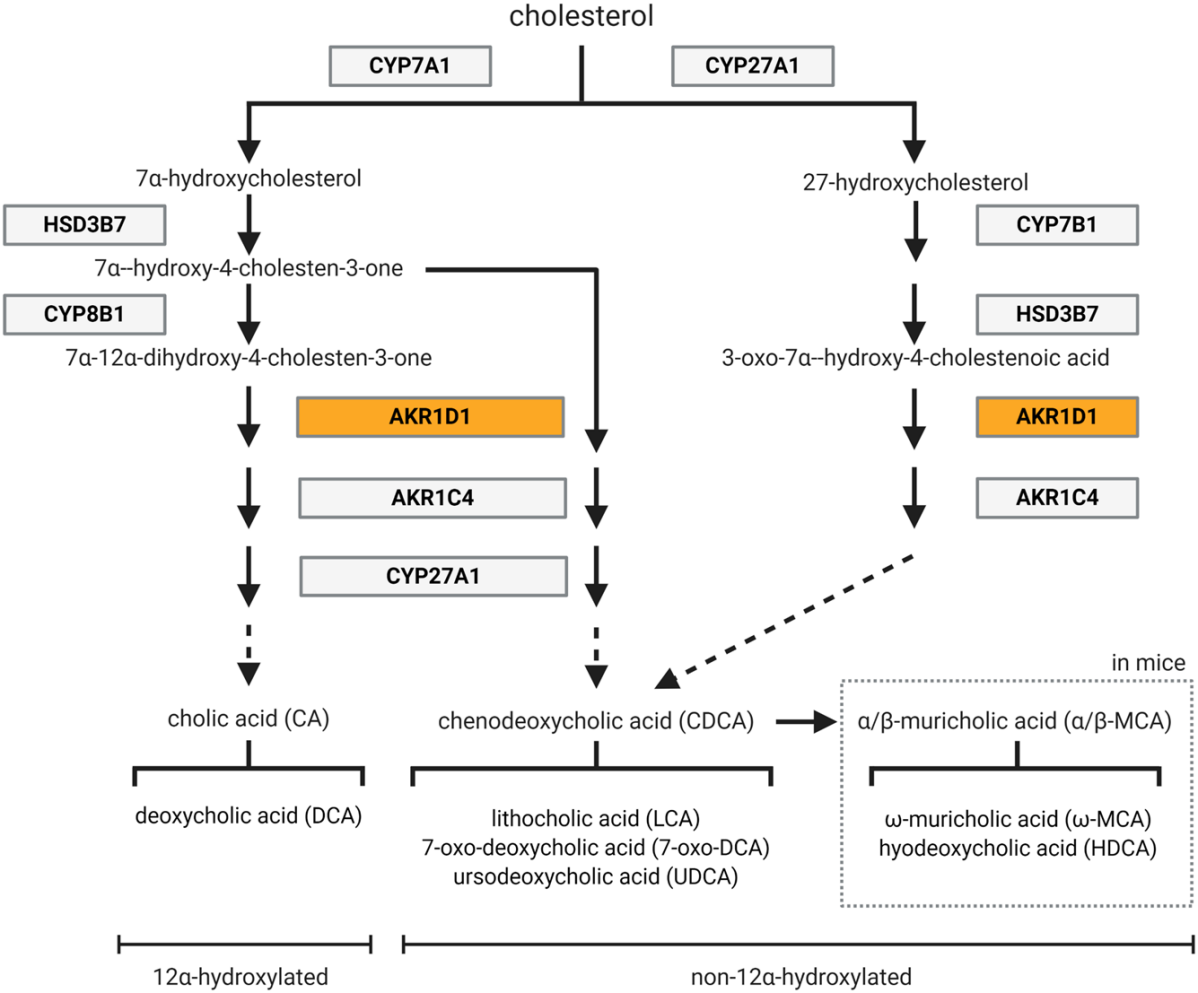
Schematic overview of the steps involved in the classic and alternative bile acid synthesis pathways in humans and mice. Figure produced in BioRender. A full colour version of this figure is available at https://doi.org/10.1530/JOE-21-0280.

Bile acids inhibit their own synthesis *via* FXR (*Nr1h4*) activation of small heterodimer partner (SHP: *Nr0b2*) downstream, repressing the expression of bile acid-synthesising enzymes. Despite lower hepatic bile acids, there was no reduction in *Shp (Nr0b2)* expression, and in *Akr1d1^–/–^*males, the expression of bile acid-synthesising enzymes was unchanged (Fig. 1E). In females, the expression of *Cyp7a1* and *Cyp8b1* was increased, with only the latter reaching significance (Fig. 1E). The intermediates of the classic pathway, 7α-12α-dihydroxy-4-chol-3-one (Fig. 1F) and 7α-hydroxy-4-chol-3-one (Fig. 1G), were increased, and there was a trend towards decreased 27-hydroxycholesterol levels (Fig. 1H), the first metabolite in the alternative pathway suggesting an increase in *Cyp7a1* activity. The bile acid synthesis pathway is presented in Fig. 2. In addition to altering synthesis, the expression of genes involved in bile acid detoxification was increased in *Akr1d1^–/–^* females. This included the phase I (oxidation) genes, *Cyp3a11, Cyp2c55* and *Cyp4a12a,* as well as the phase II (conjugation) gene *Sult2a7* (Fig. 1I). Changes in expression of key regulated genes were confirmed by qPCR (Supplementary Table 2). Sulfated bile acid species were not measured by LC-MS, but consistent with increased bile acid detoxification and clearance in *Akr1d1^–/–^* females, serum T-lithocholate-3- sulphate was increased (Fig. 1J). Consistent with this gene expression pattern in females, IPA (upstream regulators) predicted activation of the key transcriptional regulators of cholesterol metabolism, constitutive androstane receptor (CAR: *Nr1i3*) and PXR (*Nr1i2*) (Table 1A).

**Table 1:** Top 10 upstream regulators in *Akr1d1^–/–^* liver predicted by ingenuity pathway analysis (IPA). IPA of liver RNA-Seq identified core metabolic transcription factors as upstream regulators of the hepatic response to *Akr1d1* deletion. In mature (30 weeks) females, IPA predicted activation of PXR and CAR signalling (A). In mature (30 weeks) males, IPA predicted activation of STAT5B and RXRα signalling and inhibition of PPARα and PPARγ signalling (B). Activation z-score infers activation status of predicted regulators. Overlap *P*  value measures overlap between the data set genes and genes known to be regulated by the transcriptional regulator. Analysis was performed on RNA-Seq data from *n*  = 10 mice.

Upstream regulator	Molecule type	z-score	Bias-corrected z-score	*P* value of overlap	Downregulated target molecules	Upregulated target molecules
(A) Female						
Triadimefon	Chemical toxicant	1.452	0.888	3.00E-11	Hsd3b4 (includes others)	Ces2c, CYP2C18, Cyp3a25 (includes others), CYP3A5, CYP8B1
POR	Enzyme			3.68E-11	Hsd3b4 (includes others)	Aldh1a7, Ces2a, Ces2c, CYP2C18, CYP8B1, MSMO1, UGDH
NR1I3	Ligand-dependent nuclear receptor	1.955	1.29	2.08E-07		Aldh1a7, Ces2a, Ces2c, CYP3A5, CYP8B1
2,4,5,2’,4’,5’-hexaclorobhenyl	Chemical toxicant	2.236	1.954	2.28E-07		Ces2c, CYP2C18, Cyp3a25 (includes others), CYP3A5, UGDH
RORC	Ligand-dependent nuclear receptor			1.32E-06	Hsd3b4 (includes others)	Cyp3a25 (includes others), CYP3A5, CYP8B1
NR1I2	Ligand-dependent nuclear receptor	2.18	1.974	1.53E-06	Hsd3b4 (includes others)	Aldh1a7, Ces2a, Ces2c, CYP3A5
Phenobarbital	Chemical toxicant	1.675	1.206	3.33E-06		Ces2a, CYP2C18, CYP3A5, CYP8B1
RORA	Ligand-dependent nuclear receptor			3.61E-06	Hsd3b4 (includes others)	Cyp3a25 (includes others), CYP3A5, CYP8B1
1,4-bis[2-(3,5-dichloropyridyloxy)]benzene	Chemical toxicant	1.695	1.242	1.33E-05		Ces2a, Ces2c, CYP3A5, CYP8B1
Pregnenolone carbonitrile	Chemical drug			1.49E-05		Ces2a, CYP2C18, CYP3A5
(B) Male						
NFE2L2	Transcription regulator	−1.246	−0.034	7.42E-09	ABCC3, ATF3, Cyp4a14, GSTA5, PPARG, SLC7A11, SRXN1	BGLAP, NUCB2, Cyp2a12/Cyp2a22, SAA1, SERPINA3
TNF	Cytokine	0.267	1.101	8.58E-08	ABCC3, ADORA1, ATF3, CBR3, CCL22, CIDEC, GPRC5B, H19, LY6D, MMP12, PLIN4, PPARG, SLC16A5, TOX	BGLAP, IL1R1, NUCB2, Orm1 (includes others), PRTN3, SAA1, SERPINA3
STAT5B	Transcription regulator	2.345	2.524	2.19E-07	ALDH3A2, CORIN, NT5E, TOX, PDZRN3, SLC16A5, SYBU, VNN1	Cyp2a12/Cyp2a22
1,2-dithiol-3-thione	Chemical reagent	−0.017	1.135	2.37E-07	ABCC3, Cyp4a14, GSTA5, SRXN1	Cyp2a12/Cyp2a22, NUCB2, SAA1, SERPINA3
Pirinixic acid	Chemical toxicant	−2.946	−1.932	9.74E-07	ABCC3, ALDH3A2, CIDEC, Cyp4a14, LY6D, PLIN4, PPARG, SLC16A5	Orm1 (includes others), SAA1
PPARA	Ligand-dependent nuclear receptor	−1.544	−1.125	1.31E-06	ALDH3A2, CIDEC, Cyp4a14, LY6D, PLIN4, PPARG, VNN1	Orm1 (includes others), SAA1, SELENBP
PPARG	ligand-dependent nuclear receptor	−2.178	-1.751	2.31E-06	CCL22, CIDEC, CORIN, Cyp4a14, LY6D, PLIN4, PPARG, VNN1	BGLAP, SAA1
RXRA	Ligand-dependent nuclear receptor	2.200	2.449	3.71E-06	ABCC3, CCL22, MMP12, PPARG, SEMA4D	BGLAP, Cyp2c70, Orm1 (includes others),
TFRC	Transporter	1.342	0.337	5.62E-06	ATF3, MMP12, PPARG, SRXN1	PRTN3
Ciprofibrate	Chemical drug	−1.709	−1.764	5.72E-06	Cyp4a14, LY6D, PPARG, SLC22A25	Orm1 (includes others), SELENBP

Contrasting with the marked impact on bile acid homeostasis, *Akr1d1* deletion did not alter serum glucocorticoid levels or glucocorticoid-regulated gene expression in the liver. Adrenal mass (Supplementary Fig. 1B) and serum corticosterone levels (the major circulating rodent glucocorticoid) (Supplementary Fig. 1C) were unchanged. Consistent with no change in glucocorticoid receptor activation in the liver, hepatic expression of the glucocorticoid-regulated genes, serum and glucocorticoid-regulated kinase 1 (*Sgk1*), glucocorticoid-induced leucine zipper protein-1 (*GLIZ: Tsc22d3*), dual specificity phosphatase 1 (*Dusp1*), as well as the glucocorticoid metabolizing enzymes 5αR1 & 2 (*Srd5a1* & *2*), 11β-HSD1 (*Hsd11b1),*3α-hydroxysteroid dehydrogenase (*Akr1c6*) and 20α-hydroxysteroid dehydrogenase (*Akr1c18*), was unchanged (Supplementary Fig. 1D). Serum levels of other steroid substrates/products of AKR1D1, including testosterone (Supplementary Fig. 1E) and dihydrotestosterone (Supplementary Fig. 1F), were not altered.

In contrast to patients with AKR1D1 deficiency, *Akr1d1^–/–^* mice did not show overt signs of cholestasis (Supplementary Fig. 2A), hepatic inflammation (Supplementary Fig. 2B) or liver damage (Supplementary Fig. 2C and D).

### Mature (30 week) *Akr1d1^–/–^* males, but not females, have reduced fat mass and improved insulin tolerance

**Figure 3:**
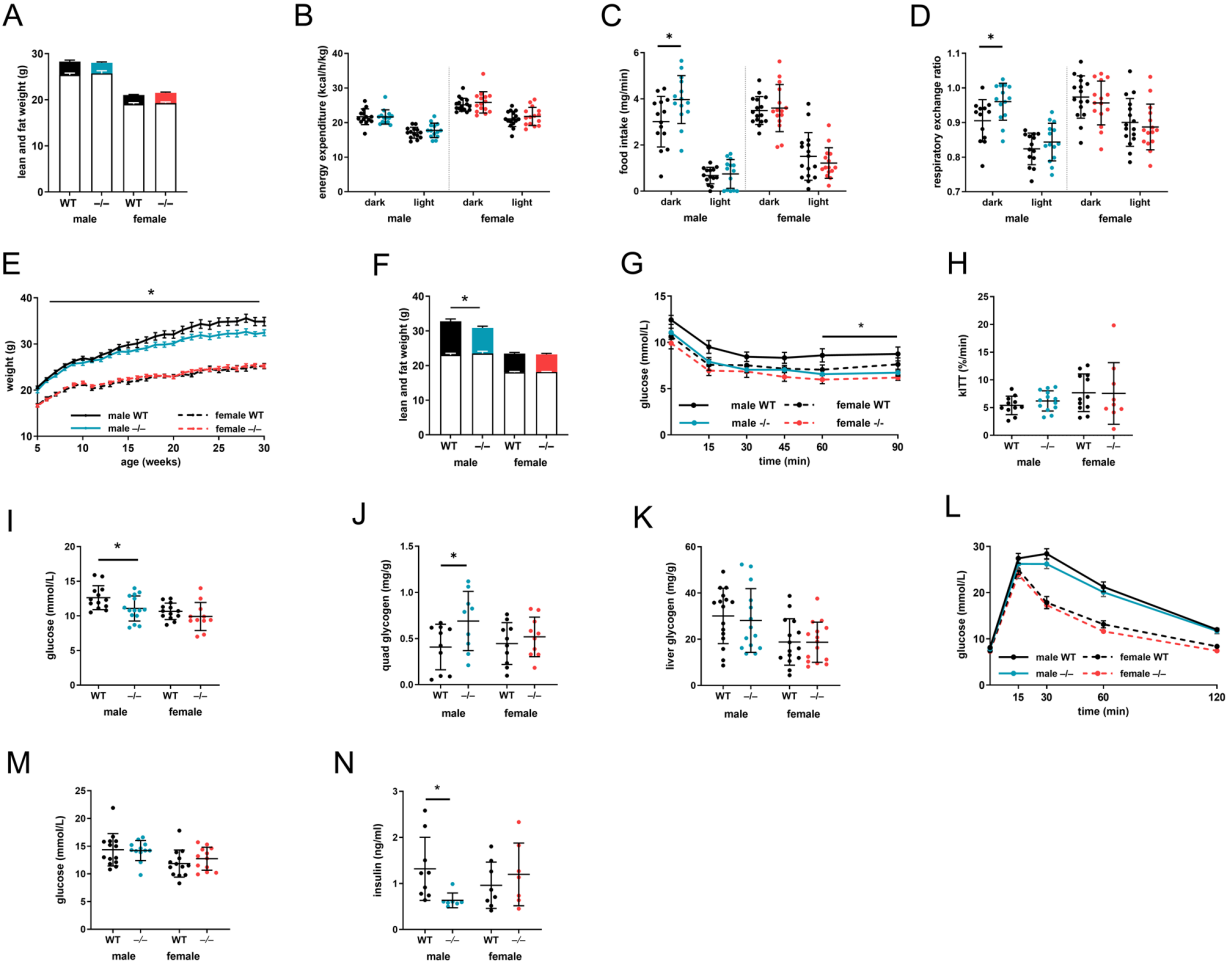
At maturity, male *Akr1d1^–/–^* mice have reduced fat mass and improved insulin tolerance. Young (10-week) *Akr1d1^–/–^* mice have normal body weight and composition (lean mass empty bar; fat mass filled bar) (A) and energy expenditure (B) is unchanged (*n*  = 14–16). Male, but not female, *Akr1d1^–/–^* mice have increased dark phase food intake (C) and a preference for carbohydrate over lipid as an energy source, measured as an increase in respiratory exchange ratio (D) (*n*  = 14–16). Male (blue lines), but not female (red lines), *Akr1d1^–/–^* mice gain less weight than their WT (black line) littermates (E) and at 30 weeks have lower fat mass without change in lean mass (F) (lean mass empty bar; fat mass filled bar) (*n*  = 14–16). *Akr1d1^–/–^* males have exaggerated glucose clearance after i.p. insulin injection (G), normal 30 min kITT (H), reduced blood glucose after a 4-h fast (I) and reduced quadricep (J) (*n*  = 11–15), but normal liver, glycogen (K). Although i.p. glucose tolerance (L) and fed serum glucose (M) are comparable to WT animals (*n*  = 11–15), fed insulin concentration is decreased (N) (*n*  = 7–8). Data are presented as mean ± s.d. *P  < 0.05, **P  < 0.01 compared to WT of the same sex. (WT = WT C57BL/6; –/– = *Akr1d1^–/–^*). A full colour version of this figure is available at https://doi.org/10.1530/JOE-21-0280.

Metabolic assessments were undertaken in young mice (10 weeks) as well as at maturity (30 weeks). At 10 weeks, body weight and composition of *Akr1d1^–/–^* mice were comparable to WT littermates (Fig. 3A). Energy expenditure (Fig. 3B) and activity rates (Supplementary Fig. 3A) were unchanged, but male *Akr1d1^–/–^* mice had a 32% increase in dark phase food intake (Fig. 3C) and a higher respiratory exchange ratio (RER), suggesting increased preference for carbohydrates over lipids as an energy source (Fig. 3D). Fecal energy (Supplementary Fig. 3B) and lipid content (Supplementary Fig. 3C) were normal, suggesting no increase in energy loss through malabsorption. *Akr1d1^–/–^* males gained weight at a slower rate than WT littermates (Fig. 3E) and were 7.5% lighter at 30 weeks of age, with dual-energy X-ray absorptiometry (DEXA) body composition analysis showing a 26% decrease in fat mass without change in lean mass (Fig. 3F).

At 10 weeks, glucose control was normal, with no change in insulin tolerance (Supplementary Fig. 3D), ipGTT or OGTT (Supplementary Fig. 3E and F), serum GLP-1 15 min post-oral glucose bolus (Supplementary Fig. 3G) or in serum insulin 60 min post i.p. glucose (Supplementary Fig. 3H). In contrast to the 10-week cohort, mature (30 weeks) male *Akr1d1^–/–^* mice had enhanced insulin tolerance as measured across an insulin tolerance test (Fig. 3G), although glucose disposal rate across the first 30 min (kITT) was unchanged (Fig. 3H). Consistent with this finding, fasting glucose was reduced in *Akr1d1^–/–^* males in response to a 4-h fast (Fig. 3I), however, not after an 18-h overnight fast (male WT 8.11 ± 0.27 vs *^–/–^* 7.87 ± 0.26 mmol/L; female WT 7.42 ± 0.24 vs *^–/–^* 7.31 ± 0.23 mmol/L). Furthermore, quadricep muscle glycogen was increased in *Akr1d1^–/–^* males (Fig. 3J), although liver glycogen remained unchanged (Fig. 3K). Despite improved insulin tolerance, ipGTT was unchanged (Fig. 3L) as was fed blood glucose (Fig. 3M). Circulating insulin levels were reduced in fed *Akr1d1^–/–^* males (Fig. 3N), suggesting a compensatory reduction in insulin secretion.

In contrast to male mice, *Akr1d1^–/–^* females had normal food intake (Fig. 3C), gained weight at the same rate as WT littermates (Fig. 3E), had normal body composition (Fig. 3F), insulin tolerance (Fig. 3G and H), quadricep muscle glycogen (Fig. 3J), fasting glucose (Fig. 3I) and fed insulin (Fig. 3N).

### *Akr1d1^–/–^* males have reduced hepatic and adipose lipid stores and hypertriglyceridemia

**Figure 4:**
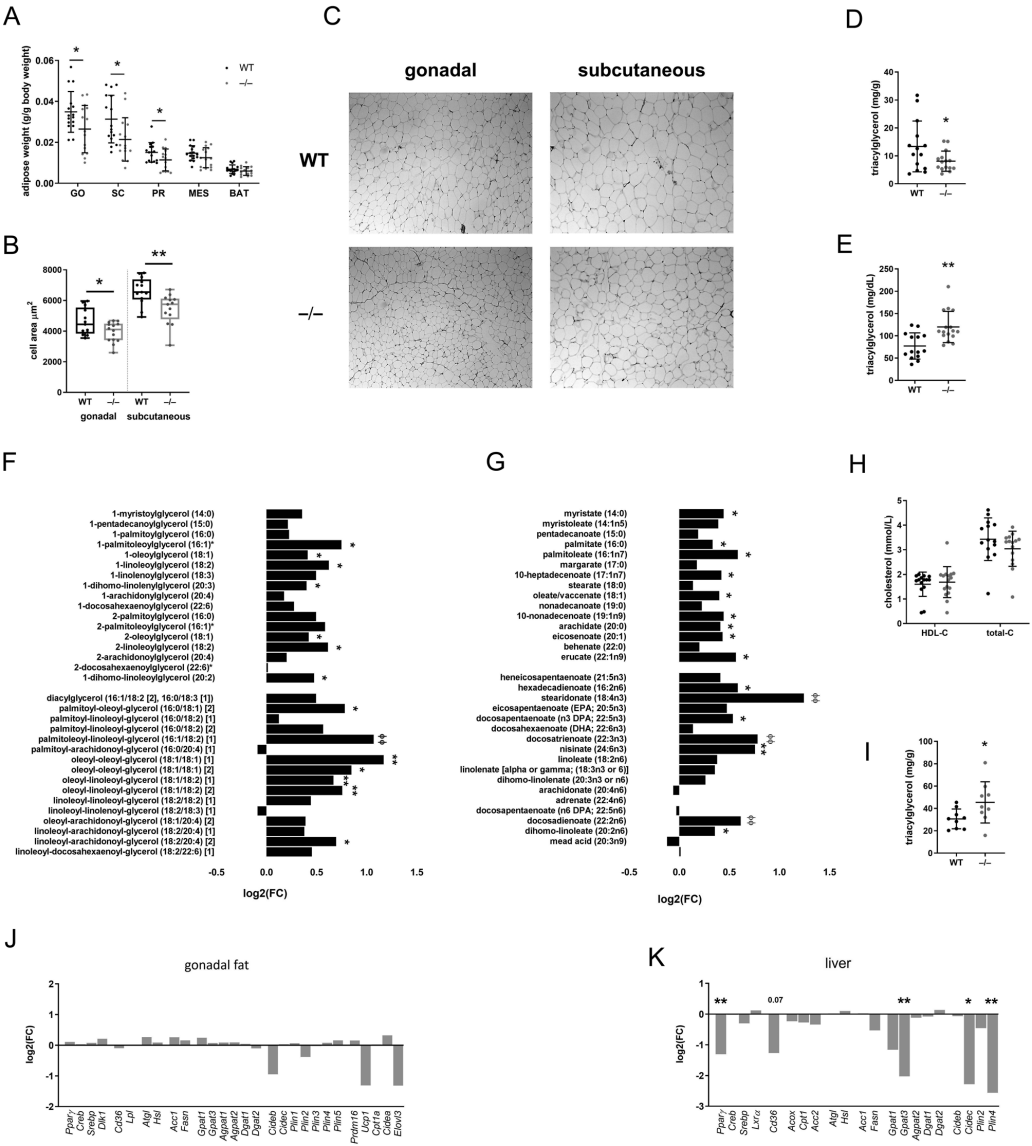
Male *Akr1d1^–/–^* mice have reduced adipose and hepatic lipid accumulation and hypertriglyceridemia. Mature (30 week) male *Akr1d1^–/–^* mice (grey points) have smaller gonadal, subcutaneous and peri-renal adipose tissue weights compared to WT littermates (black points) (A) (*n*  = 14–15 mice) with smaller adipocytes in the gonadal and subcutaneous depots (B and C) (*n*  = 13–14 mice) and reduced hepatic triacylglycerol (D) (*n*  = 14–15 mice). Serum triacylglycerol (E) (*n*  = 14–16), monoacylglycerols and diacylglycerols (F) and non-esterified fatty acids (G) are increased, but total and HDL cholesterol (H) are normal (*n*  = 10 mice). Intra-muscular triacylglycerol is increased in *Akr1d1^–/–^* quadricep muscle (I) (*n*  = 9 mice). The mRNA expression of lipid metabolism genes in the gonadal fat is unchanged (J), but in the liver, the expression of fatty acid esterification (*Gpat3*) and lipid droplet (*Cidec* & *Plin4*) genes as well as the transcription factor *Pparg* are reduced (K) (*n*  = 10 mice). *P  < 0.05, **P  < 0.01, ***P  < 0.005, ^∅^ *P*  < 0.001, ^∅∅^ *P*  < 0.0005 compared to wildtype (WT = WT C57BL/6; –/– = *Akr1d1^–/–^*).

Male *Akr1d1^–/–^* mice had reduced gonadal, subcutaneous and peri-renal adipose depot weights (Fig. 4A), and adipocytes were smaller in the gonadal and subcutaneous depots (Fig. 4B and C). Furthermore, hepatic triacyclglycerol accumulation was reduced in *Akr1d1^–/–^* males (Fig. 4D). *Akr1d1^–/–^* males had increased serum triacyclglycerols (Fig. 4E), monoacylglycerols and diacylglycerols (Fig. 4F) and non-esterified fatty acids (Fig. 4G), but without change in total or HDL cholesterol (Fig. 4H). Relative intensity values for acylglycerols and fatty acids are presented in Supplementary Table 3. Hypertriglyceridemia is commonly associated with increased intramyocellular triacylglycerol accumulation, and skeletal muscle triacylglycerol levels were increased in the *Akr1d1^–/–^* males (Fig. 4I).

Despite hypertriglyceridemia and reduced adipose mass, there was no change in the expression of key lipid metabolism genes in the gonadal fat from *Akr1d1^–/–^* males (Fig. 4J), and IPA (causal network) did not predict altered lipid accumulation. In the liver, genes involved in fatty acid uptake (*Cd36*, *P* = 0.07), esterification (*Gpat3*) and lipid storage (*Cidec* & *Plin4*) (Fig. 4K) were decreased, and IPA (causal network) predicted reduced lipid accumulation. Consistent with reduced lipid accumulation, IPA (upstream regulators) predicted PPARγ inhibition (Table 1B). In the quadricep muscle, the expression of key genes involved in the regulation of lipid and carbohydrate metabolism was unchanged (Supplementary Table 2). In *Akr1d1^–/–^* females, total fat mass was unchanged (Fig. 3B), but gonadal, subcutaneous and peri-renal adipose depots were smaller (Supplementary Fig. 4A), though to a lesser degree than in males. Serum total and HDL cholesterol (Supplementary Fig. 4B), total serum triacylglycerol (Supplementary Fig. 4C), diacylglycerol and monoacylglycerol (Supplementary Fig. 4D) were all normal, but levels of some non-esterified fatty acids were reduced (Supplementary Fig. 4E). Hepatic triacyclglycerol (Supplementary Fig. 4F) content was unchanged. Relative intensity values for acylglycerols and fatty acids are presented in Supplementary Table 3. The expression of key lipid metabolism genes was not altered in the gonadal fat (Supplementary Fig. 4G) or liver (Supplementary Fig. 4H).

### Male *Akr1d1^–/–^* mice are not protected against diet-induced obesity or insulin resistance

**Figure 5:**
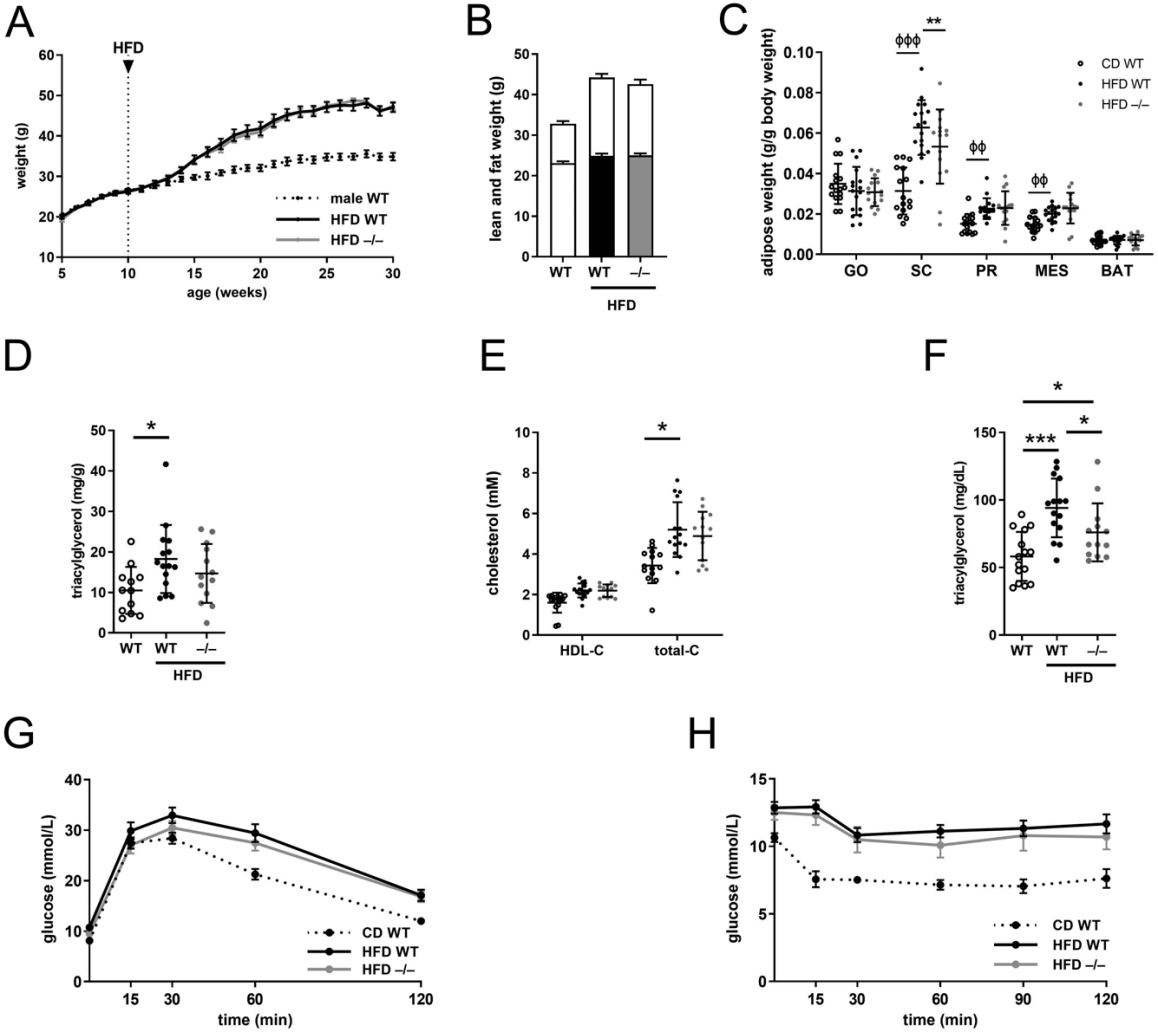
*Akr1d1* deletion does not protect male C57BL/6 mice against diet-induced obesity, hypercholesterolemia or insulin resistance but improves hypertriglyceridemia. On a high fat diet (HFD), male *Akr1d1^–/–^* mice (grey line) gain weight at the same rate as their WT littermates (black line) (A) and after 20 weeks body composition is not different between genotypes (lean mass lower bar; fat mass upper bar) (B) (*n*  = 14–15 mice). Adipose weight (C) (*n*  = 14–15), hepatic triacylglycerol (D) (*n* = 12–15), HDL and total cholesterol (E) (*n*  = 14–15) were unchanged between HFD fed WT and *Akr1d1^–/–^* males. Serum triacylglycerol was reduced in HFD fed *Akr1d1^–/–^* males but not to levels seen in the control diet (F) (*n*  = 10 mice). *Akr1d1^–/–^* males were not protected against diet-induced reduction in ipGTT (G) or ipITT (H) (*n*  = 14–15 mice). Data are presented as mean ± s.d.*P  < 0.05, **P  < 0.01, ***P  < 0.005 compared to wildtype (WT = WT C57BL/6; –/– = *Akr1d1^–/–^*).

To investigate the interaction between genotype and diet, 10-week-old male mice were challenged with a high fat diet (HFD) (60% kcal from fat) for 20 weeks. Figures include WT control data to allow comparison. On HFD,* Akr1d1^–/–^* males gained weight at the same rate as WT littermates (Fig. 5A), and body composition (Fig. 5B), adipose weights (Fig. 5C), hepatic triacylglycerol (Fig. 5D), and total and HDL cholesterol (Fig. 5E) were unchanged. Male *Akr1d1^–/–^* mice were partially protected against diet-induced hypertriglyceridemia (Fig. 5F) but not glucose intolerance (Fig. 5G) or insulin resistance (Fig. 5H).

**Figure 6:**
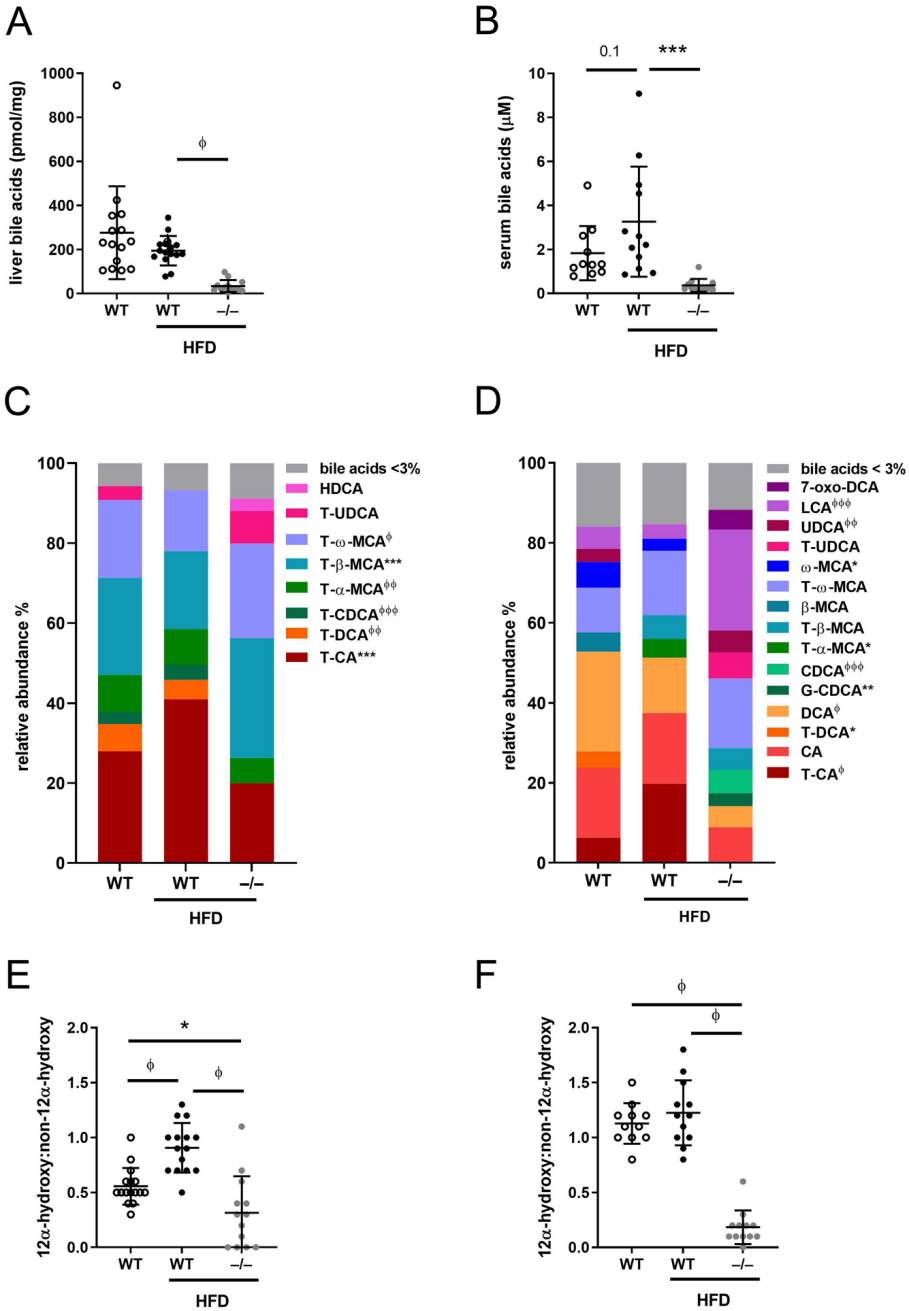
Bile acid profile in male *Akr1d1^–/–^* mice on a high fat diet (HFD). After 20 weeks on a HFD, male *Akr1d1^–/–^* mice have reduced total liver (A) and serum (B) bile acids and altered bile acid composition (liver: C; serum D) compared to high fat-fed WT littermates. Serum 12α-hydroxylated/non-12α-hydroxylated bile ratio in the liver (E) and serum (F) is reduced compared to control and HFD fed WT littermates. Data are presented as mean ± s.d. in *n*  =10–15 mice. *P  < 0.05, **P  < 0.01, ***P  < 0.005, ^∅^ *P*  < 0.001, ^∅∅^ *P*  < 0.0005, ^∅∅∅^ *P*  < 0.0001. WT control diet data are presented for comparison, *P* value for bile acid composition compares WT and *Akr1d1^–/–^* on HFD (WT = WT C57BL/6; –/– = *Akr1d1^–/–^*). A full colour version of this figure is available at https://doi.org/10.1530/JOE-21-0280.

Total hepatic bile acids were reduced in WT mice on HFD but trended towards an increase in the serum (Fig. 6A and B). *Akr1d1* deletion reduced total bile acids in both liver (Fig. 6A) and serum (Fig. 6B). Bile acid composition was altered (liver: Fig. 6C; serum: Fig. 6D), and the 12α-hydroxylated/non-12α-hydroxylated bile acid ratio reduced (liver: Fig. 6E; serum: Fig. 6F). Absolute levels of liver and serum bile acids are presented in Supplementary Table 4.

## Discussion

Here we present the first *in vivo* evidence that AKR1D1 regulates metabolism, demonstrating a sex-specific metabolic phenotype in *Akr1d1^–/–^* mice where males, but not females, have altered lipid homeostasis and improved insulin tolerance. These effects are associated with sexually dimorphic changes in bile acid metabolism and composition of the bile acid pool.

Sitting at the interface of steroid hormone and bile acid metabolism, AKR1D1 has the potential to affect metabolic homeostasis by altering steroid hormone and/or bile acid availability. Despite its central position, only a small number of studies have investigated AKR1D1 in the context of metabolic disease; hepatic gene expression is decreased in patients with type 2 diabetes (Valanejad *et al.* 2018) and non-alcoholic fatty liver disease (Nikolaou *et al.* 2019*b*) although in one study systemic 5β-reductase activity was increased in patients with hepatic steatosis (Westerbacka *et al.* 2003). Whether reduced AKR1D1 activity contributes to the pathogenesis of metabolic disease is almost entirely unexplored, however, we have recently shown that manipulating AKR1D1 alters glucocorticoid and bile acid regulation of metabolism and inflammation *in vitro* (Nikolaou *et al.* 2019*a*, *b*, 2020).

AKR1D1 is the only known 5β-reductase for C19-C27 steroids, and patients with missense mutations in AKR1D1 produce only trace amounts of 5β-reduced bile acids (Palermo *et al.* 2008). In contrast, *Akr1d1^–/–^* mice still produce 5β-reduced bile acids, albeit at a lower level. This suggests the possibility of a second, yet unknown, 5β-reductase in mice and that the *Akr1d1^–/–^* mouse represents partial 5β-reductase deficiency and this needs to be considered when extrapolating rodent data into the human context. Patients with AKR1D1 deficiency develop severe hepatic cholestasis (Clayton *et al.* 1996), however, we saw no evidence of cholestasis or liver damage in *Akr1d1^–/–^* mice. During cholestasis, damage is caused by the accumulation of hydrophobic bile acids, in particular glyco-CDCA (Dilger *et al.* 2012), whereas, in mice, CDCA is converted to hydrophilic MCA species, protecting against intrahepatic cholestasis (Hohenester *et al.* 2020), further emphasising the potential for species-specific differences.

The mechanisms underpinning the metabolic phenotype of the *Akr1d1^–/–^* mouse are complex but do not appear to reflect glucocorticoid excess. Mice generate 5β-reduced glucocorticoid metabolites (Shackleton *et al.* 2008), and AKR1D1 controls glucocorticoid availability and action in human hepatoma cell lines (Nikolaou *et al.* 2019*a*); nevertheless, circulating corticosterone levels were normal in *Akr1d1^–/–^* mice and hepatic expression of glucocorticoid-regulated genes was unchanged. Furthermore, the observed reduction in hepatic triacylglycerol and enhanced insulin tolerance contrast with the hepatic steatosis and insulin resistance that occur in models of tissue-specific glucocorticoid excess (5αR1 deletion and hepatic 11βHSD1 overexpression) (Paterson *et al.* 2004, Dowman *et al.* 2013, Livingstone *et al.* 2015).

Male *Akr1d1^–/–^* mice exhibit lipodystrophy with reduced lipid accumulation in adipose and liver as well as hypertriglyceridemia and high serum free fatty acids. This phenotype may reflect a loss of FXR and TGR5 signalling in metabolic tissues. Consistent with this hypothesis, FXR^–/–^ mice also have reduced adipose tissue depot weights, hypertriglyceridemia and increased serum fatty acids (Cariou *et al.* 2006). The hyperlipidemia is thought to be, at least in part, due to reduced FXR stimulated adipocyte differentiation (Rizzo *et al.* 2006, Abdelkarim *et al.* 2010) and adipose lipid accumulation (Cariou *et al.* 2006). In contrast, TGR5 stimulates beige remodelling of white adipose tissue (Velazquez-Villegas *et al.* 2018); however, loss of beige remodelling in *Tgr^–/–^* mice is only evident on cold -exposure (Velazquez-Villegas *et al.* 2018). Despite reduced adiposity, RNA-Seq analysis did not identify any gene expression patterns associated with reduced adipocyte differentiation, lipid accumulation or beige remodelling. In the liver, FXR inhibits VLDL synthesis (Watanabe *et al.* 2004) and *Tgr5^–/–^*mice have increased hepatic VLDL secretion as well as decreased hepatic fatty acid uptake. It is therefore possible that loss of hepatic FXR and TGR5 signalling in *Akr1d1^–/–^* males could increase VLDL synthesis and reduce fatty acid uptake, simultaneously reducing hepatic triacylglycerol levels and contributing to hyperlipidemia. Interestingly, *Tgr5^–/–^* mice have increased skeletal muscle fatty acid uptake (Donepudi *et al.* 2017), which is consistent with the increased intramuscular triacylglycerol in *Akr1d1^–/–^* males. This may be a consequence of changes in metabolic flux due to hyperlipidemia, but the role of skeletal muscle TGR5 in regulating lipid metabolism is unexplored. RNA-Seq analysis of male *Akr1d1^–/–^* liver showed downregulation of PPARγ as well as transcriptional targets of PPARγ that are involved in fatty acid uptake and lipid storage (*Cd36*, *Gpat3*, *Cidec*, and *Plin4*). FXR is a transcriptional activator of PPARγ (Torra *et al.* 2003) and *Tgr5^–/–^* mice also have decreased *Cd36* expression (Donepudi *et al.* 2017), although the IPA did not identify wider hepatic gene expression signatures associated with FXR or TGR5 signalling. To better understand the impact of AKR1D1 deletion on the pathways involved in lipid flux, a study comparing the fasted and fed state is required.

The secondary bile acid, DCA, is a satiety signal (Wu *et al.* 2020) and the lower DCA levels seen in *Akr1d1^–/–^* males may contribute to the observed increase in food intake. Despite this increase in food intake, and without changes in lipid absorption or other measures of energy expenditure, *Akr1d1^–/–^* males still gained less weight. Bile acids, and the bioactive intermediates of their synthesis, have pleiotropic effects, and it is therefore plausible that the discrepancy between food intake and weight gain does not depend on standard measures of energy balance.

Male *Akr1d1^–/–^* mice had improved insulin tolerance, lower fasting serum glucose normal fed serum glucose despite lower serum insulin and increased quadricep glycogen, together suggesting enhanced insulin sensitivity. Insulin tolerance tests are largely a measure of glucose uptake into muscle, and the quadricep muscles had increased intramuscular triacylglycerol, usually associated with insulin resistance (Krssak *et al.* 2000). Indeed, FXR*^–/–^* mice have both dyslipidemia and insulin resistance (Cariou *et al.* 2006). The negative impact of intramuscular triacylglycerol on insulin sensitivity is dependent on its subcellular localisation (Kahn *et al.* 2021), and it is possible that intramuscular triacylglycerol is safely stored in *Akr1d1^–/–^* males. As in *Akr1d1^–/–^* mice, *Tgr5^–/–^* mice have a sexually dimorphic metabolic phenotype where (on normal chow) males, but not females, have improved insulin tolerance (Vassileva *et al.* 2010). The mechanism behind this is not understood, and in apparent contrast, transgenic mice that overexpress TGR5 in skeletal muscle have improved insulin sensitivity (Sasaki *et al*. 2021). The ability to activate (or antagonize) bile acid receptors differs considerably between bile acid species (Fiorucci & Distrutti 2015) meaning that in addition to total bile acids levels, the composition of the bile acid pool is important. A reduction in the ratio of serum 12α-hydroxylated to non-12α-hydroxylated bile acids is associated with insulin sensitivity (Haeusler *et al.* 2013), although the mechanism that underpins this is not understood. Rodent studies suggest the relationship is independent of total bile acid levels: *Cyp8b1^–/–^* and *Cyp7a1^–/–^* mice have respectively high and low total bile acids, but both have a reduced serum 12α-hydroxylated/non-12α-hydroxylated ratio and improved glucose control recoverable by supplementation with CA (Kaur *et al.* 2015, Ferrell *et al.* 2016). Mirroring the *Cyp7a1^–/–^* mice (Ferrell *et al.* 2016), *Akr1d1^–/–^* mice have a reduced 12α-hydroxylated/non-12α-hydroxylated ratio on a background of low total liver and serum bile acids. However, despite the 12α-hydroxylated/non-12α-hydroxylated ratio remaining low on the HFD, *Akr1d1^–/–^* males were not protected against the diet-induced insulin intolerance.

Whilst *Akr1d1^–/–^* males had a broad metabolic phenotype, the effect on females was mild despite a similar decrease in total bile acid levels. The mechanism that underpins the sexual dimorphism is unclear, but differences in the composition of the bile acid pool may be involved. The expression and regulation of hepatic enzymes is highly sexually dimorphic (Rando & Wahli 2011) and female-specific changes in bile acid metabolism may help to limit the impact of *Akr1d1* deletion on bile acid composition. Female *Akr1d1^–/–^* mice tended to have decreased 27-hydroxycholesterol (*P* = 0.08) with significantly increased 7α-12α-dihydroxy-4-chol-3-one and 7α-hydroxy-4-chol-3-one, suggesting cholesterol is diverted from alternative, towards classic, synthesis. The upregulation of 12α-hydroxylase (CYP8B1) in *Akr1d1^–/–^* females may help to maintain the production of CA. Bile acid detoxification pathways were also increased in females, and as sulfation and subsequent renal clearance of CDCA occurs at a rate twice that of CA (Stiehl 1974, Stiehl *et al.* 1975), enhanced CDCA clearance could also protect against the relative loss of 12α-hydroxylated bile acids. Oestradiol and progesterone are potent activators of bile acid synthesis (Chico *et al.* 1996) and energy metabolism (D’eon & Braun 2002), and a limitation of our study is that we did not assess oestrus cycle stage in our female mice.

In conclusion, we have shown that AKR1D1 activity regulates insulin tolerance and lipid metabolism *in vivo* and that its effects are sexually dimorphic. Further studies are clearly warranted to explore both the mechanisms by which this occurs and the role it plays in the pathogenesis of metabolic disease.

## Supplementary Material

Supplementary Figure 1: Akr1d1 deletion does not overtly affect glucocorticoid or sex steroid metabolism. Akr1d1 deletion (A: western blot, liver), does not alter adrenal weight (B), serum corticosterone levels (C), or hepatic mRNA expression of glucocorticoid responsive genes, Sgk1, Gilz, Dusp1 and 11βhsd1 (D) (n = 10 mice). The serum levels of testosterone and dihydrotestosterone were also unchanged. Data are presented as mean ± sd of n=10-15 30 week old mice, n.d. = not detectable.

Supplementary Figure 2: Mature (30-week) Akr1d1–/– mice show no evidence of hepatic cholestasis, inflammation or damage. Liver histology (H&E) showed no evidence of cholestasis (A) or hepatic inflammation (B). Serum levels of the markers of liver damage alanine aminotransferase (ALT) (C) and AST aspartate aminotransferase (D) were unchanged. Data are presented as mean ± sd of n=10-15 mice. (WT = wildtype C57BL/6; –/– = Akr1d1–/–)

Supplementary Figure 3: Intestinal lipid absorption and glucose control are apparently normal in young (10-week) Akr1d1–/– mice. Fecal energy (A) and lipid content (B) are normal in Akr1d1–/– mice (n = 7 mice). Ip insulin tolerance (C), ip glucose tolerance (D), oral glucose tolerance (E), serum GLP-1 15 minutes post oral glucose bolus (F) and serum insulin 60 minutes post oral glucose bolus (G) were unchanged in male or female Akr1d1–/– mice (n = 15 mice). Data are presented as mean ± sd. *p<0.05 compared to wildtype of the same sex. (WT = wildtype C57BL/6; –/– = Akr1d1–/–)

Supplementary Figure 4: In female mice Akr1d1 deletion reduces adipose mass but does not result in hypertriglyceridemia. Mature (30 week) female Akr1d1–/– mice (grey bars) have smaller gonadal, subcutaneous and peri-renal adipose weights compared to WT littermates (black bars) (A) (n = 8 mice). Serum HDL and total cholesterol (B) (n = 14-16), triacylglycerol (C) (n = 13-14), monoacylglycerols and diacylglycerols (D) (n = 10) are normal in Akr1d1–/– females, but several fatty acid species are reduced (E) (n = 10 mice). Hepatic triacylglycerol levels were unchanged (F). The expression of lipid metabolism genes in the gonadal fat (G) and liver (H) were unchanged (n = 10 mice). Data are presented as mean ± sd or log2(FC). *p<0.05 compared to wildtype. (WT = wildtype C57BL/6; –/– = Akr1d1–/–)

Supplementary Material

## Declaration of interest

The authors declare that there is no conflict of interest that could be perceived as prejudicing the impartiality of the research reported.

## Funding

This work was supported by the Medical Research Council http://dx.doi.org/10.13039/501100000265, UK (programme grant awarded to J W T ref. MR/P011462/1); NIHR Oxford Biomedical Research Centre http://dx.doi.org/10.13039/501100013373 (Principal investigator award to J W T) based at Oxford University Hospitals http://dx.doi.org/10.13039/100012324 NHS Trust and University of Oxford; Nigel Groome PhD Studentship awarded to L L G and A A; Bioscientifica Trust Grant to N N; Swiss National Science Foundation http://dx.doi.org/10.13039/100000001 No 31003A-179400 (Principle Investigator A O). The views expressed are those of the authors and not necessarily those of the NHS, the NIHR or the Department of Health.

## Author contribution statement

N Nikolaou and S E Harris contributed equally to this work.
