# Supplementary figures and images for: AKR1D1 knockout mice develop a sex-dependent metabolic phenotype
Source: J Endocrinol. 2022 Mar 23;253(3):97–113. doi: 10.1530/JOE-21-0280 (PMC9086936; doi:10.1530/JOE-21-0280)

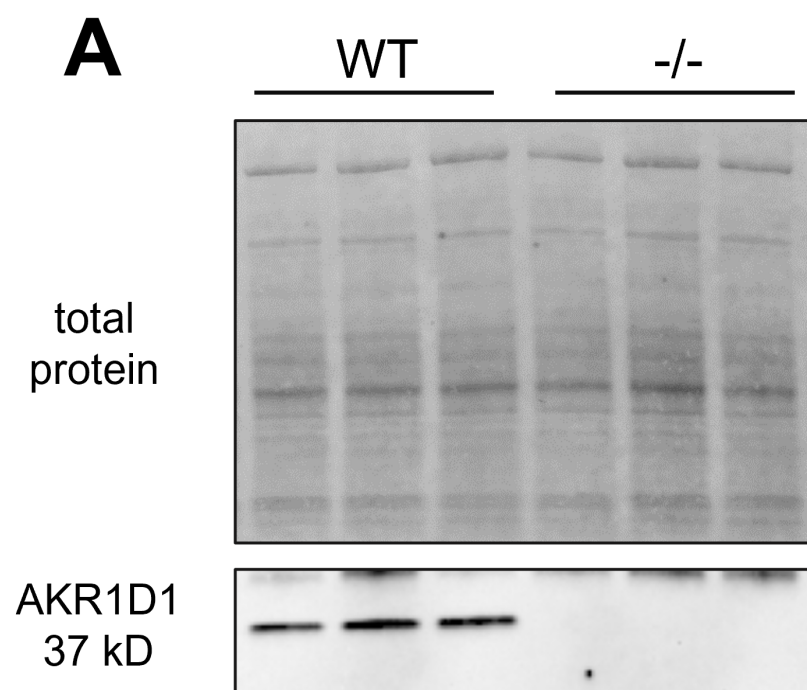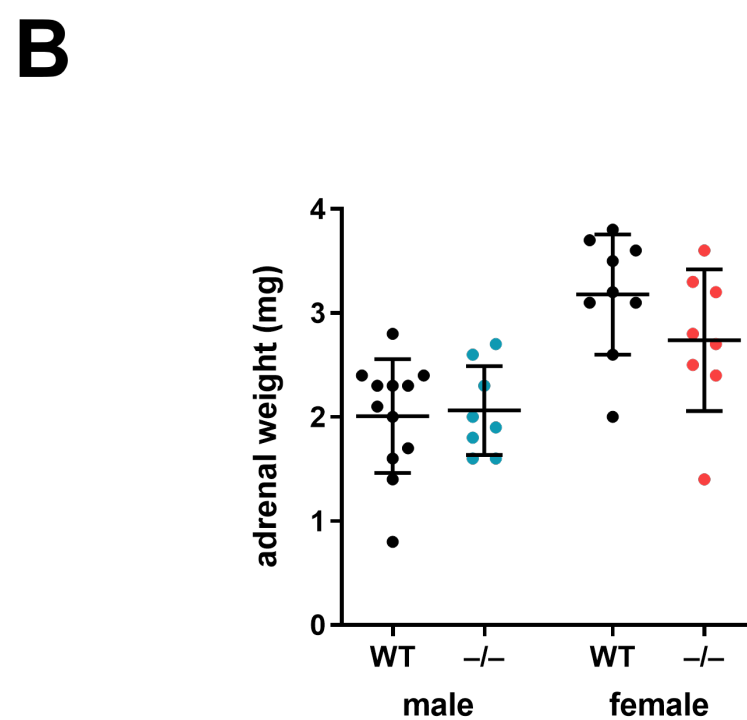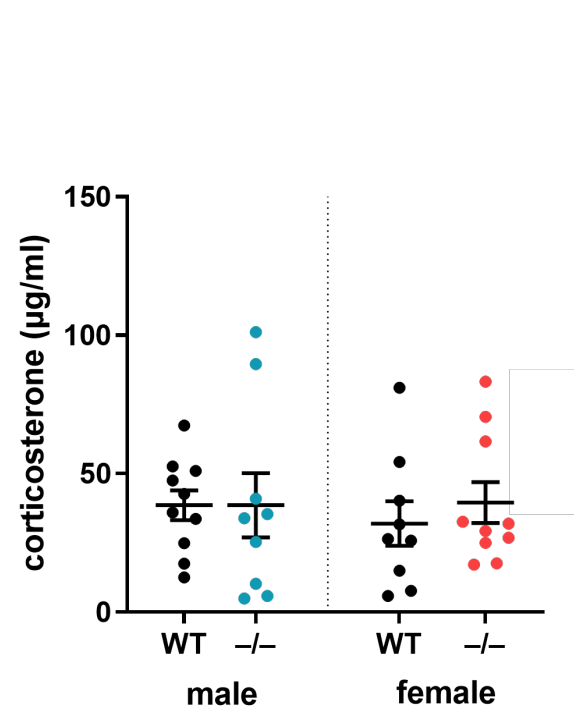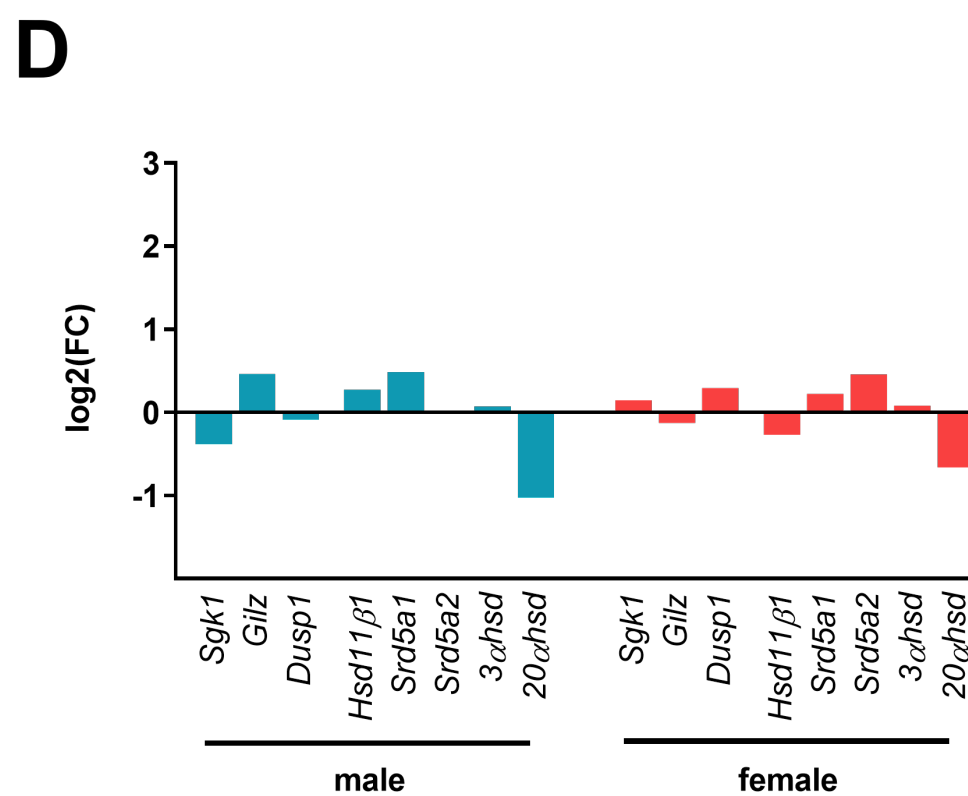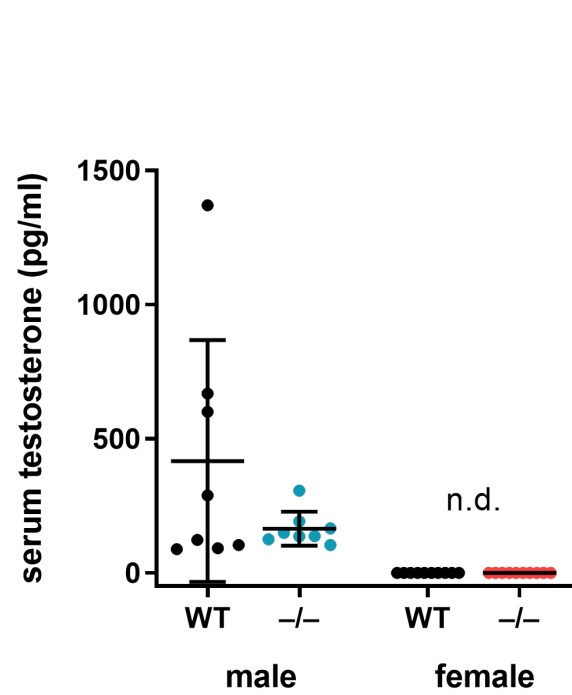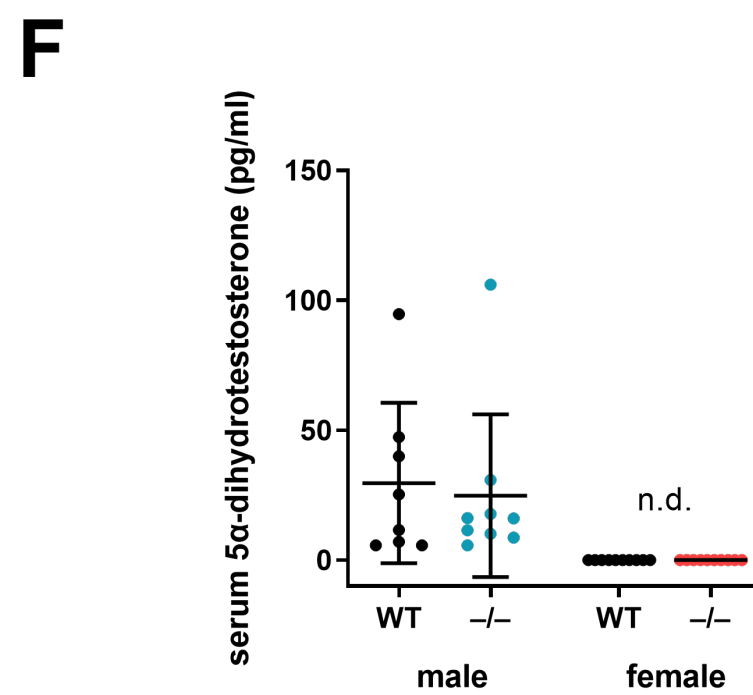

Supplement: Supplementary Figure 1: Akr1d1 deletion does not overtly affect glucocorticoid or sex steroid metabolism. Akr1d1 deletion (A: western blot, liver), does not alter adrenal weight (B), serum corticosterone levels (C), or hepatic mRNA expression of glucocorticoid responsive genes, Sgk1, Gilz, Dusp1 and [file supplementary_figure_1.pdf]

**A**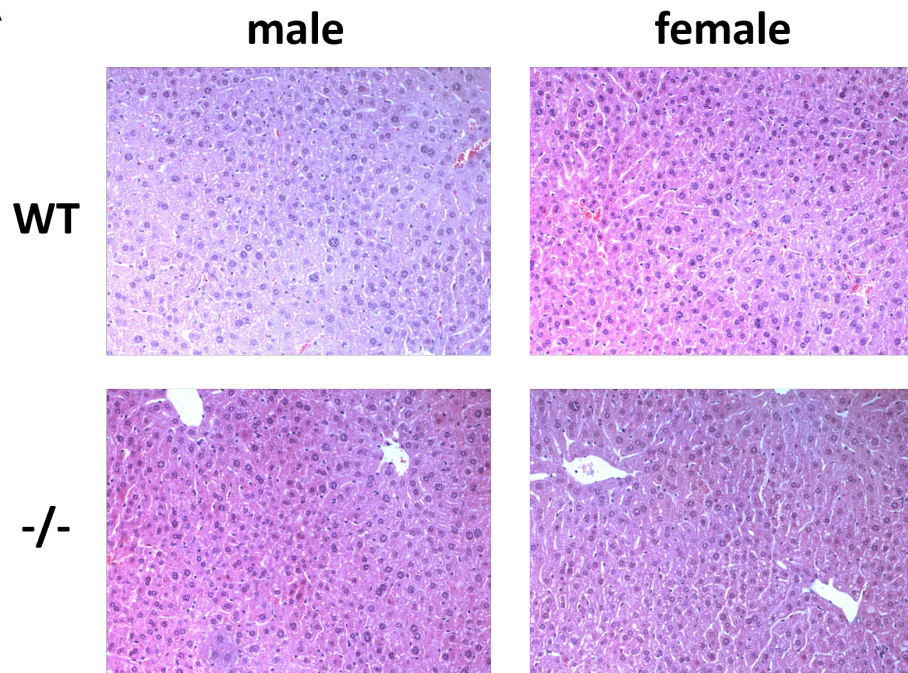**B**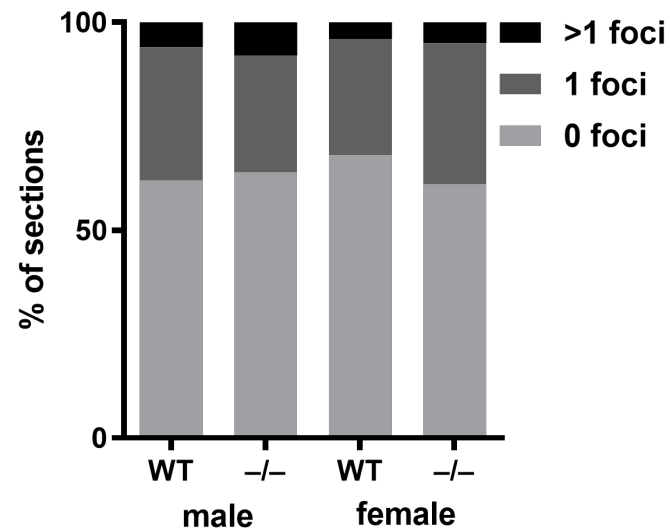**C**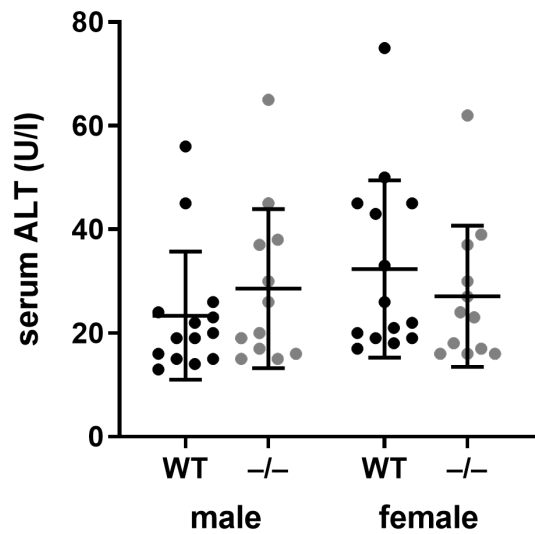**D**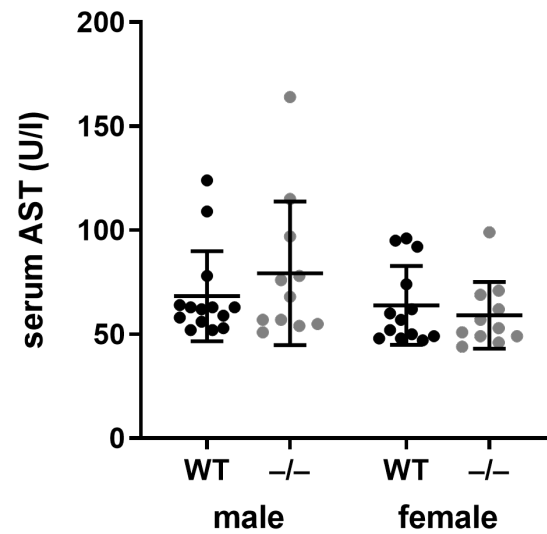

Supplement: Supplementary Figure 2: Mature (30-week) Akr1d1–/– mice show no evidence of hepatic cholestasis, inflammation or damage. Liver histology (H&E) showed no evidence of cholestasis (A) or hepatic inflammation (B). Serum levels of the markers of liver damage alanine aminotransferase (ALT) (C) and AST asp [file supplementary_figure_2.pdf]

**A**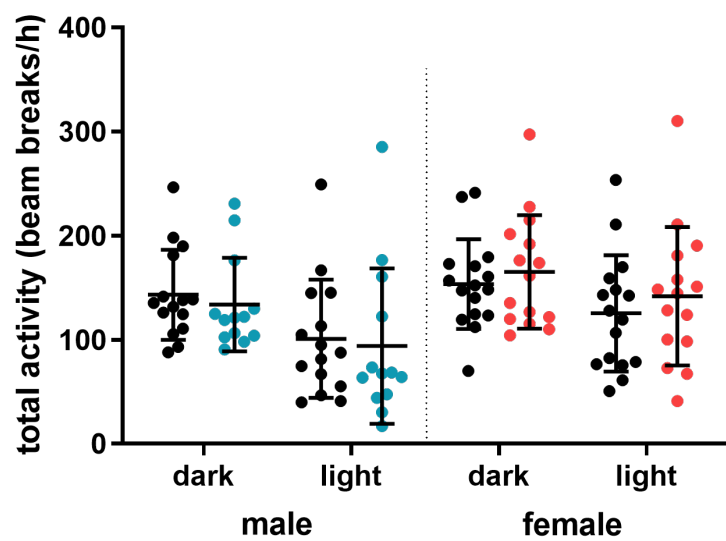**B**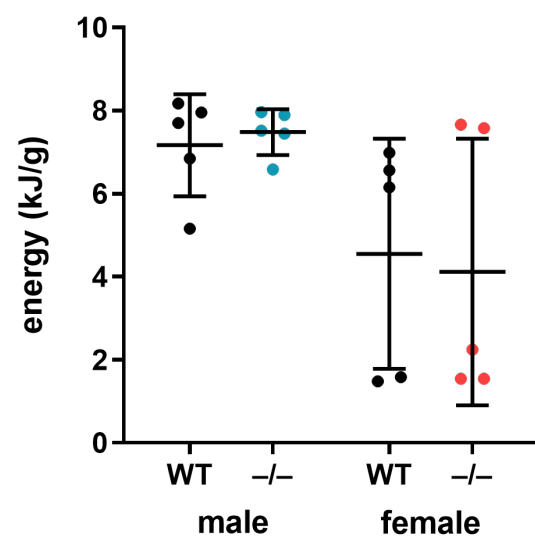**C**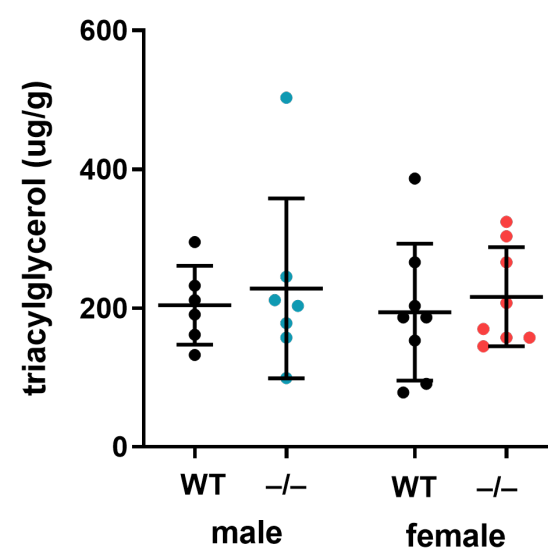**D**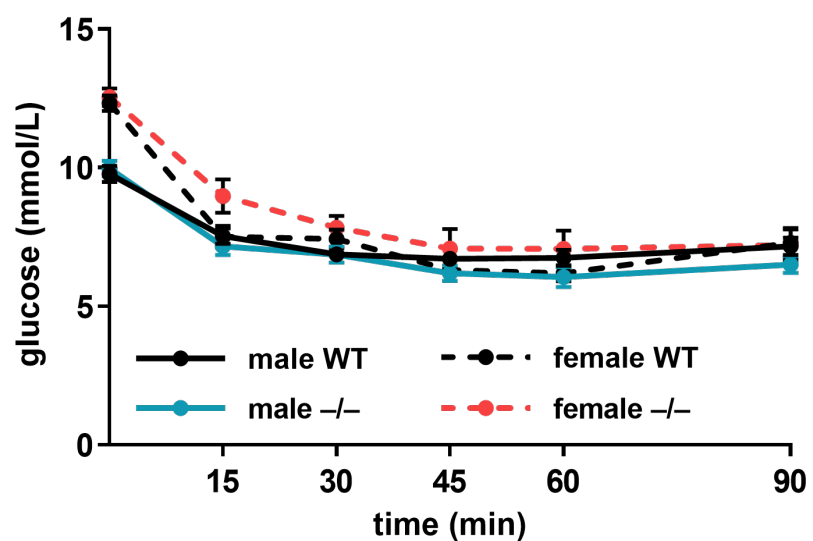**E**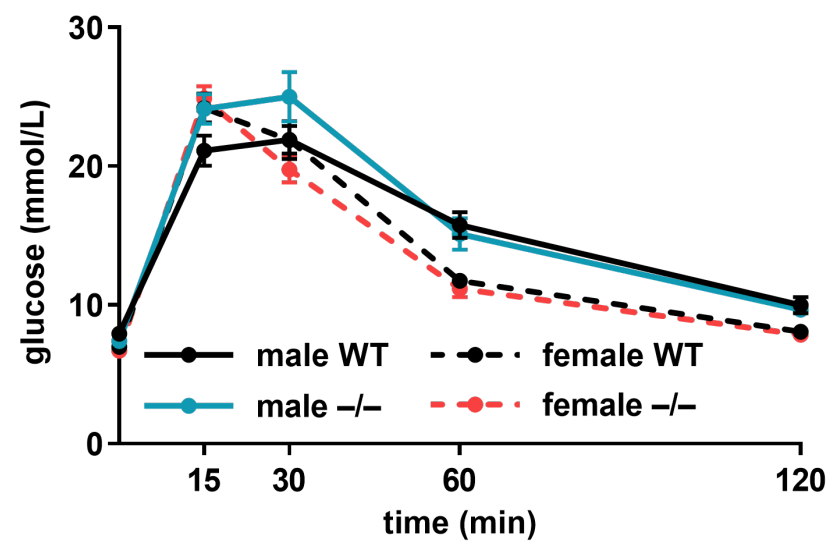**F**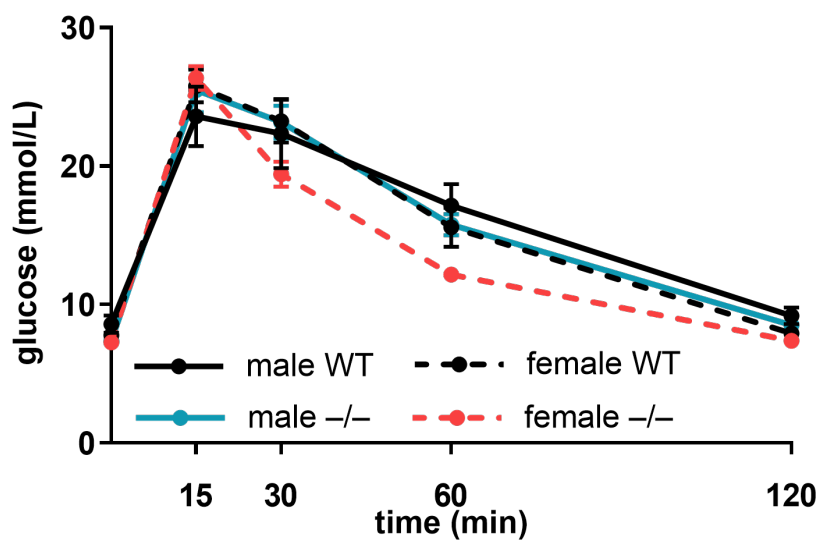**G**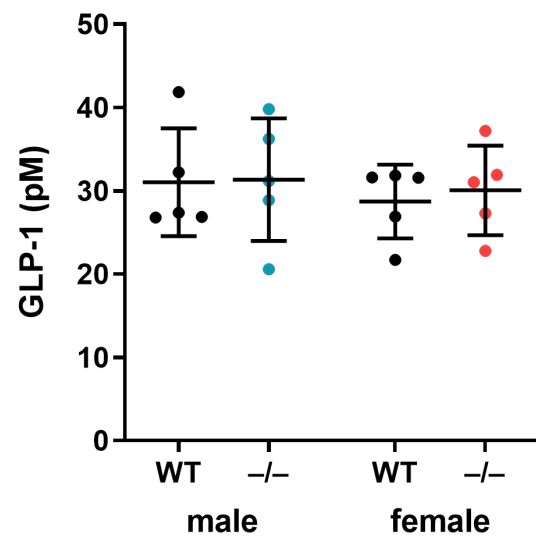**H**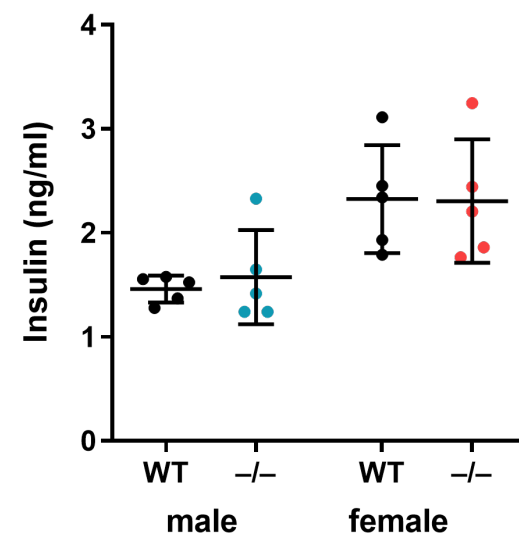

Supplement: Supplementary Figure 3: Intestinal lipid absorption and glucose control are apparently normal in young (10-week) Akr1d1–/– mice. Fecal energy (A) and lipid content (B) are normal in Akr1d1–/– mice (n = 7 mice). Ip insulin tolerance (C), ip glucose tolerance (D), oral glucose tolerance (E), serum GLP [file supplementary_figure_3.pdf]

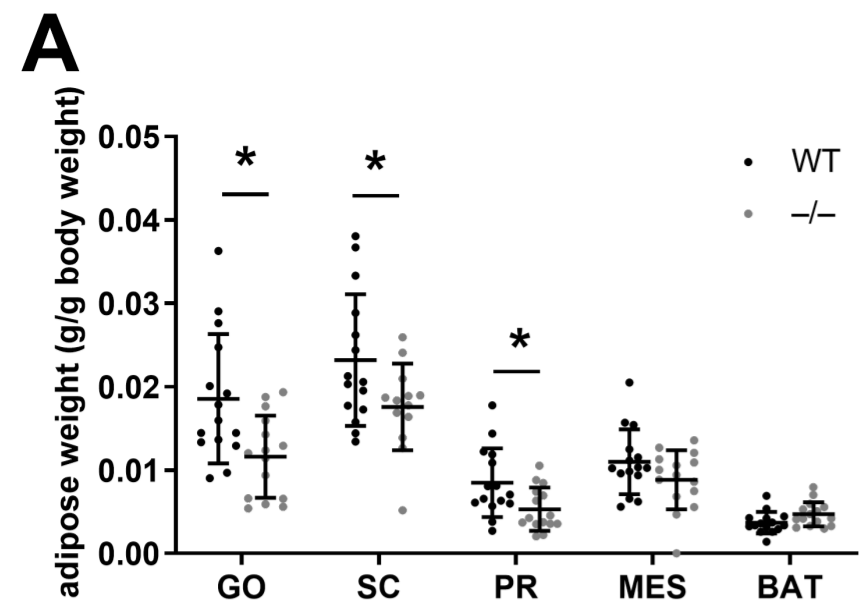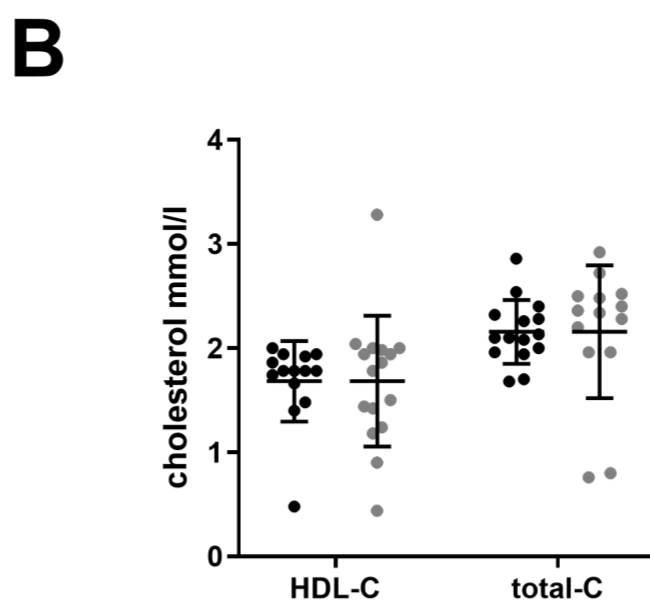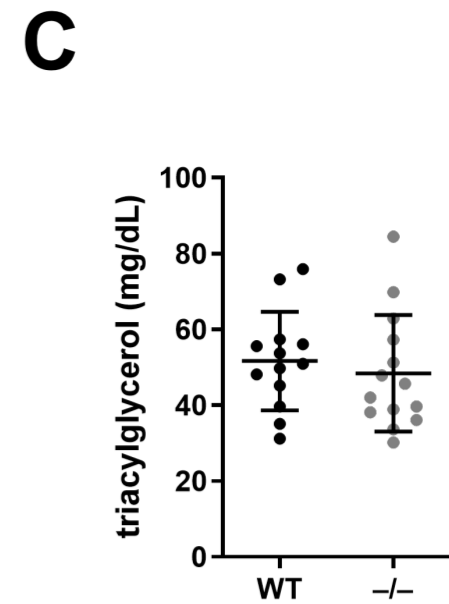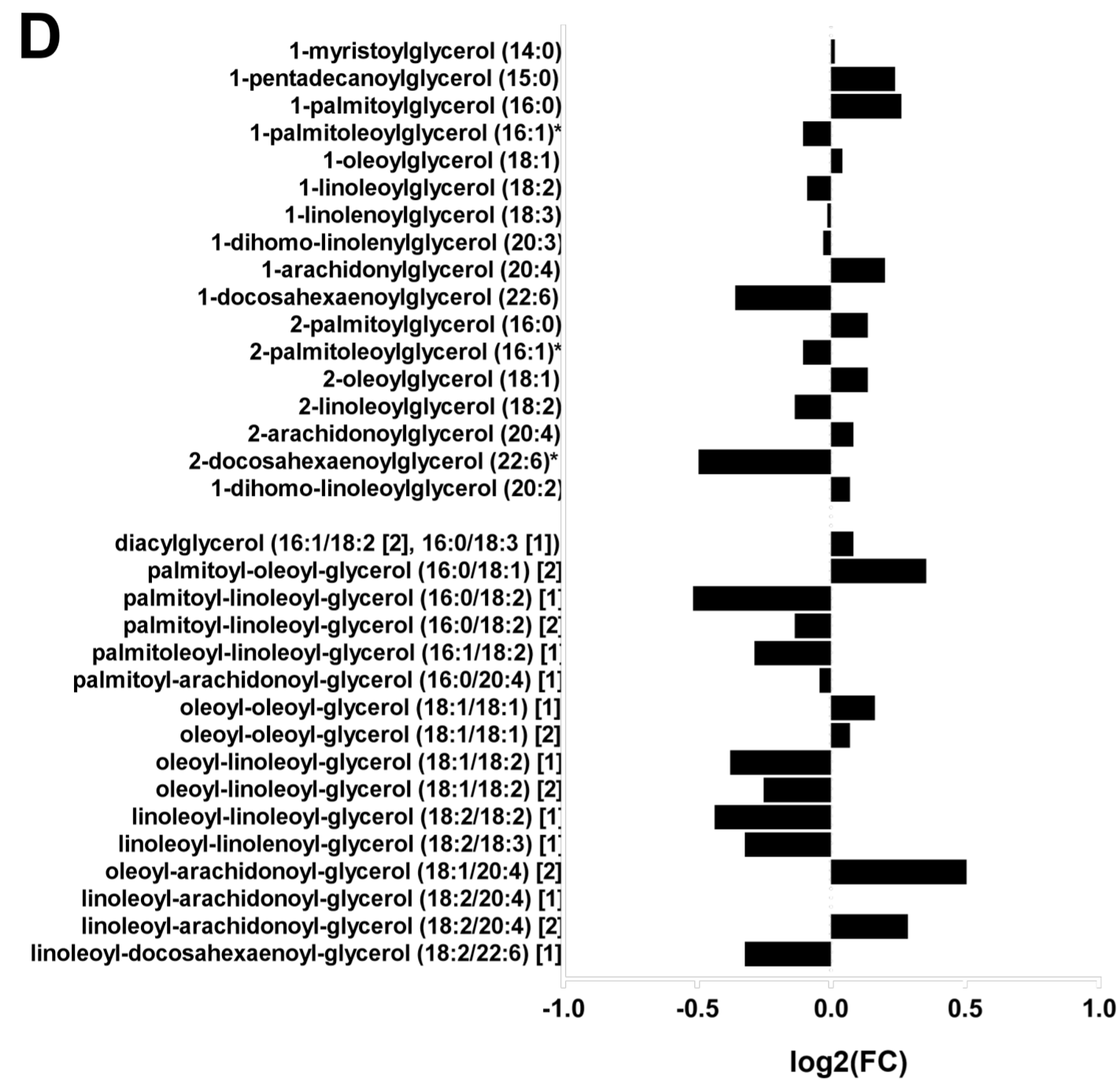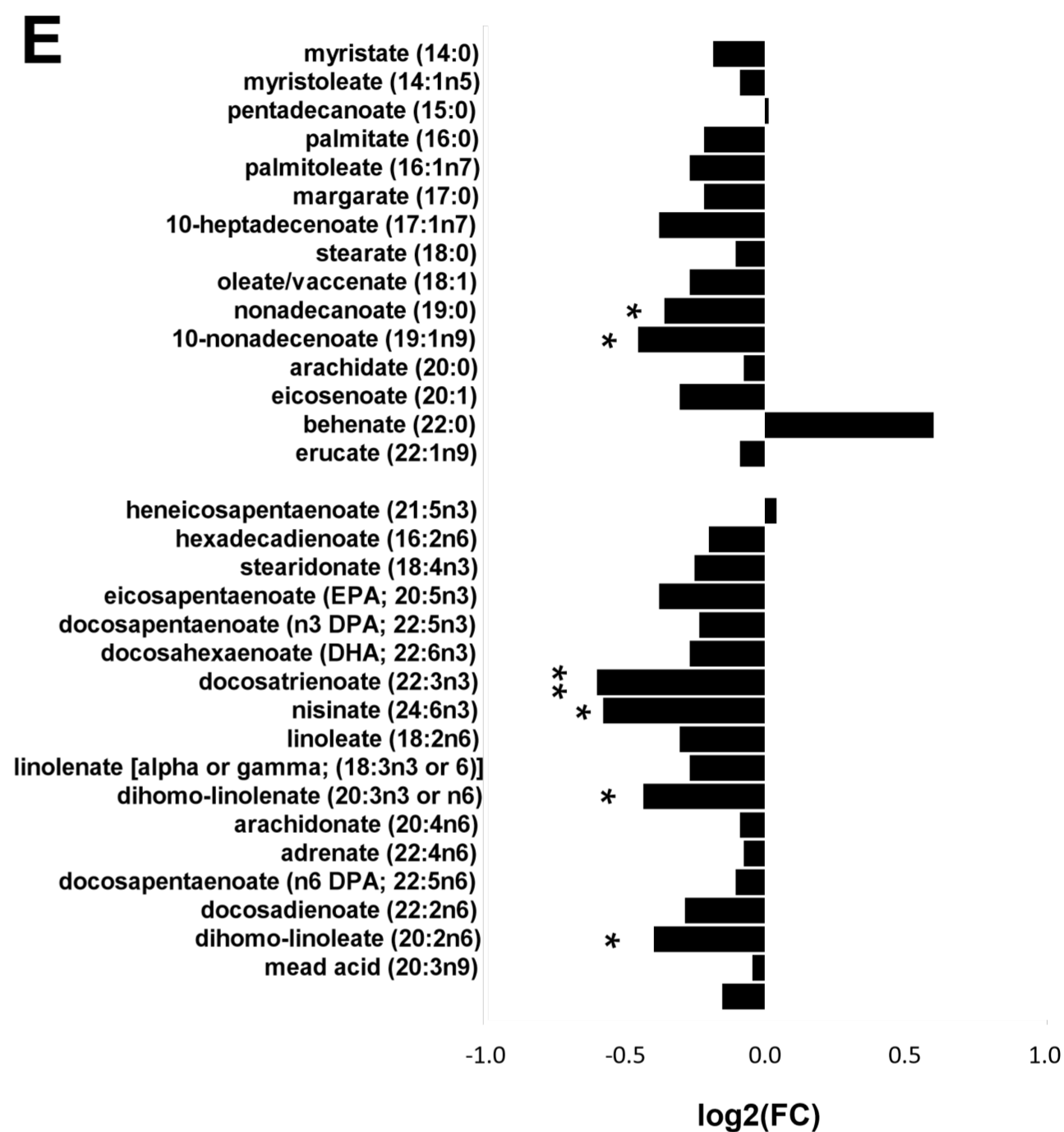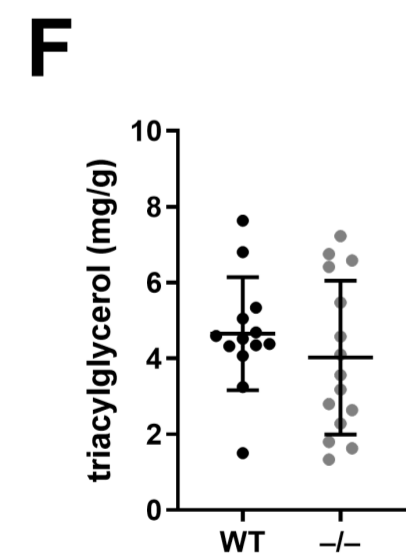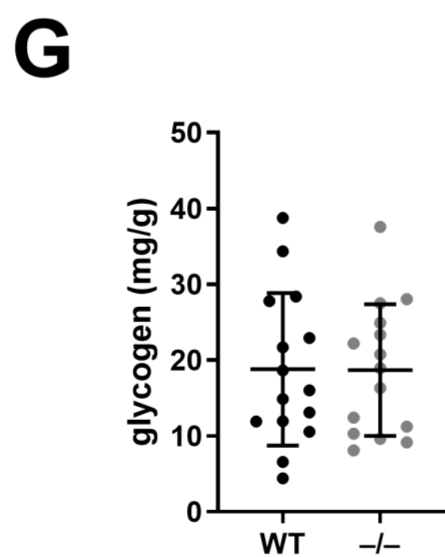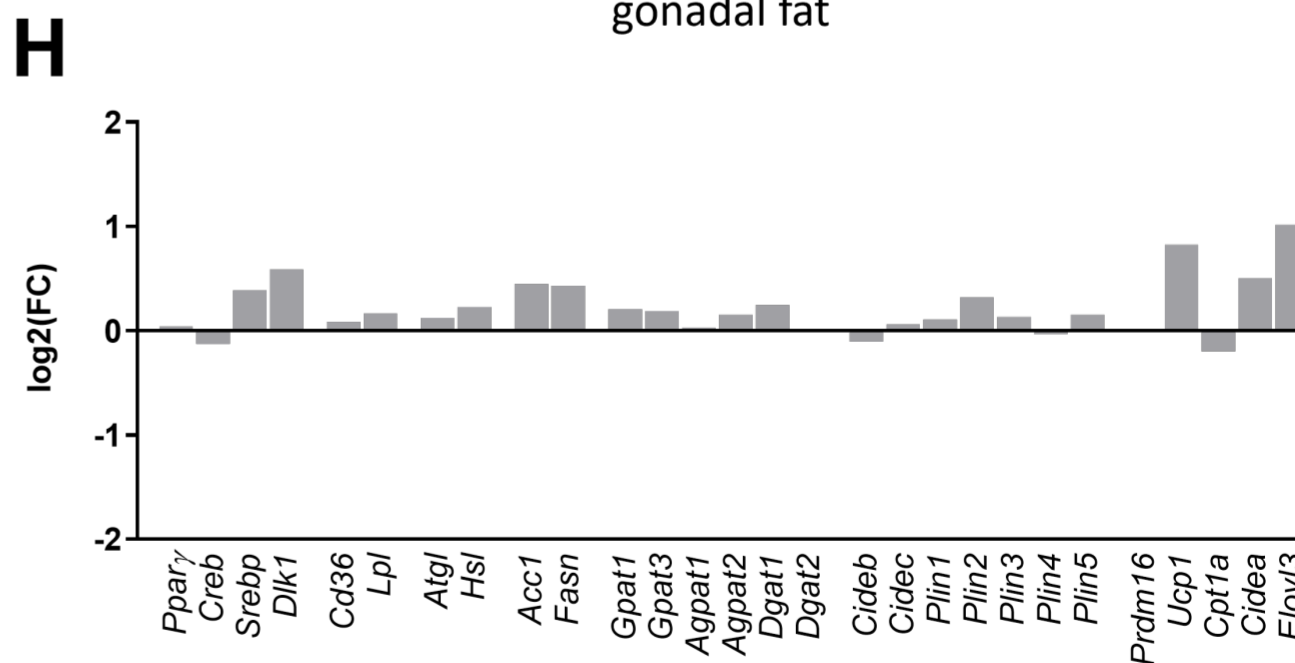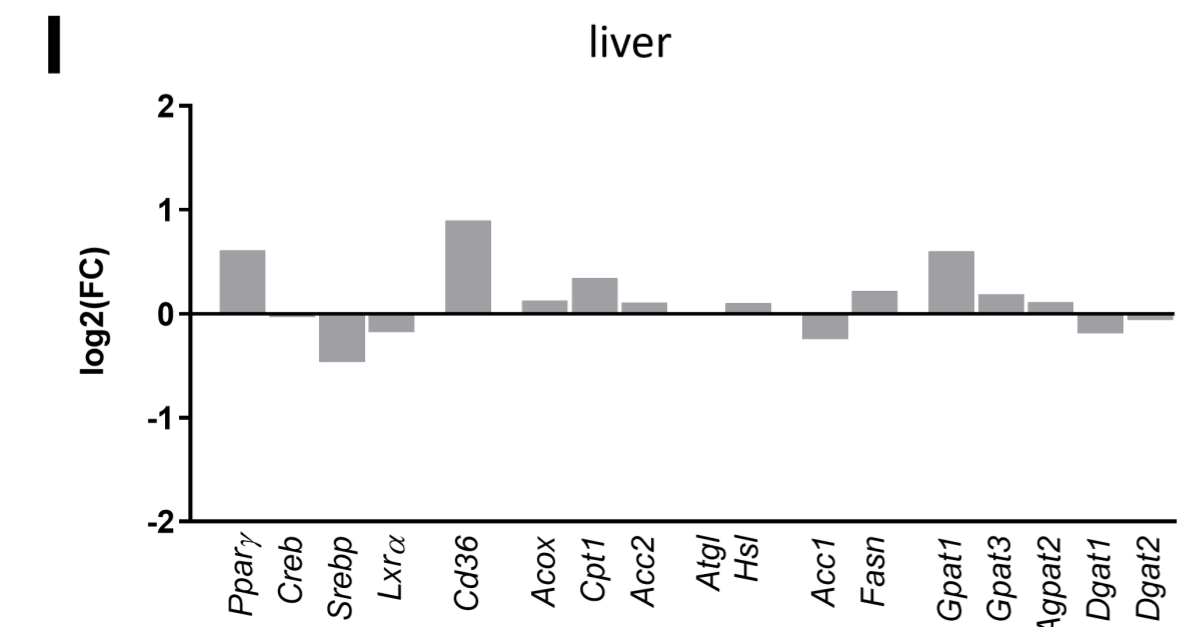

Supplement: Supplementary Figure 4: In female mice Akr1d1 deletion reduces adipose mass but does not result in hypertriglyceridemia. Mature (30 week) female Akr1d1–/– mice (grey bars) have smaller gonadal, subcutaneous and peri-renal adipose weights compared to WT littermates (black bars) (A) (n = 8 mice). Seru [file supplementary_figure_4.pdf]
