# Supplementary material for: AKR1D1 knockout mice develop a sex-dependent metabolic phenotype
Source: J Endocrinol. 2022 Mar 23;253(3):97–113. doi: 10.1530/JOE-21-0280 (PMC9086936; doi:10.1530/JOE-21-0280)
Supplement: Supplementary Material [file supplementary_material.pdf]

Supplementary Table 1. Liver and serum bile acid concentrations in mature (30-week) wildtype and *Akr1d1*$^{-/-}$ mice on control diet. Plasma bile acid concentration in liver and serum of wildtype and *Akr1d1*$^{-/-}$ mice. *p<0.05, **p<0.01, ***p<0.005, $^{\emptyset}$p<0.001, $^{\emptyset\emptyset}$p<0.0005, $^{\emptyset\emptyset\emptyset}$p<0.0001 compared to wildtype within sex. T, tauro; G, glycol; CA, cholic acid; CDCA, chenodeoxycholic acid; MCA, murocholic acid; DCA, deoxycholic acid; LCA, lithocholic acid; UDCA, ursodeoxycholic acid; HDCA, hyodeoxycholic acid; ND, not detected. Data are presented as mean ± se of n= 11-15 mice.

	**liver (pmol/mg)**				**serum (nM)**			
	**male**		**female**		**male**		**female**	
	**WT**	***Akr1d1–/–***	**WT**	***Akr1d1–/–***	**WT**	***Akr1d1–/–***	**WT**	***Akr1d1–***
**CA**	5.23±1.88	0.12±0.06	6.26±2.58	1.22±0.53	365±112	49.1.6±20.	837±177	246±90
**T-CA**	78.1±13.8	7.55±1.85$^{\phi}$	115±16.5	33.3±9.26*	92.3±16.3	5.1±5.1$^{\phi}$	417±47	143±24$^{\phi}$
**G-CA**	0.47±0.09	0.01±0.01*	0.47±0.08	0.21±0.03	ND	ND	ND	ND
**CDCA**	0.17±0.03	ND$^{\phi}$	0.09±0.02	ND	31.2±4.8	19.2±2.3	33.3±4.3	18.7±1.9
**T-CDCA**	7.32±1.66	0.62±0.13*	9.39±1.45	1.59±0.39$^{\phi}$	9.09±1.21	0.69±0.69$^{\phi}$	18.0±2.8	0.68±0.47
**G-CDCA**	ND	ND	ND	ND	6.80±1.50	9.83±2.30	11.2±1.5	9.95±1.81
**α-MCA**	0.97±0.30	0.03±0.02*	0.49±0.13	0.07±0.04	20.1±10.0	1.49±1.49	42.5±10.9	4.35±2.82
**T-α-MCA**	24.8±4.9	2.40±0.58*	27.6±4.0	4.89±1.32	46.7±10.4	7.9±1.7**	107±13	ND$^{\phi\phi\phi}$
**β-MCA**	4.87±1.20	0.85±0.24	1.62±0.35	0.54±0.14	111±39	24.1±8.1	450±114	147±47
**T-β-MCA**	74.9±13.3	18.4±3.9**	97.0±11.3	42.7±8.2*	56.4±13.9	11.36±3.7*	169±15	98±37
**ω-MCA**	1.03±0.22	0.24±0.06	0.61±0.19	0.21±0.06	129±33	31.3±6.4	398±79	113±32*
**T-ω-MCA**	60.3±12.4	20.7±4.5	67.2±9.97	32.8±6.9	190±27	62.2±11.8*	560±38	246±40$^{\phi\phi}$
**DCA**	0.64±0.20	ND	0.28±0.03	0.02±0.02$^{\phi}$	443±77	37.4±15.4$^{\phi}$	1076±131	96.4±24.4
**T-DCA**	15.4±2.2	0.77±0.18$^{\phi}$	20.0±2.6	2.35±0.66$^{\phi}$	58.7±9.9	1.83±1.5$^{\phi\phi\phi}$	156±22	3.39±2.02
**LCA**	ND	ND	ND	ND	77.5±3.7	68.7±5.8	65.8±3.1	65.5±4.8
**T-LCA**	1.47±0.07	0.99±0.24	1.85±0.13	1.94±0.22	0.65±0.16	1.21±1.04	0.69±0.27	1.35±0.38
**G-LCA**	ND	ND	ND	ND	ND	ND	ND	ND
**UDCA**	0.36±0.10	0.28±0.08	0.23±0.10	0.14±0.04	66.2±20.2	45.5±5.1	177±73	33.6±5.6
**T-UDCA**	10.1±2.1	7.49±1.23	12.8±2.01	7.77±1.94	42.6±3.75	20.0±3.4**	94.2±7.6	61.0±11.0
**G-UDCA**	0.01±0.004	ND	0.01±0.004	ND	5.62±0.2	6.35±0.64	6.04±0.15	6.23±0.15
**HDCA**	0.62±0.01	2.28±0.44*	0.08±0.03	0.18±0.05	54.9±18.2	28.4±5.1	125±21	5.38±2.13
**7-oxo-DCA**	ND	ND	0.09±0.05	0.03±0.02	20.1±13.8	40.0±11.3	25.6±11.4	140±101
**T-7-oxo-LCA**	0.10±0.04	ND	0.23±0.06	0.04±0.03	ND	ND	ND	ND
**12α-hydroxyl**	99.7±17.5	8.44±2.04	142±20	37.1±10.3	959±180	93±44	2486±299	489±113
**non-12α-**	187±34	55.3±10.4	219±27	92.9±18.5	868±178	379±44	2282±305	952±183

Supplementary Table 2. mRNA expression of bile acid metabolism, insulin signaling and lipid metabolism genes in the liver and quadricep muscle of mature (30-week) *Akr1d1*$^{-/-}$ mice. Data are presented as mean ± se of relative expression ratio, n = 9-12 mice. *p<0.05 compared to wildtype. (WT = wildtype C57BL/6; –/– = *Akr1d1*$^{-/-}$)

	**male liver relative expression ratio**		**female liver relative expression ratio**	
	**WT**	***Akr1d1–/–***	**WT**	***Akr1d1–/–***
Cyp7a1	0.37±0.08	0.25±0.08	1.62±0.41	2.51±0.80
Cyp8b1	0.93±0.10	0.69±0.05	0.60±0.15	1.34±0.21*
Cyp3a11	0.59±0.11	0.63±0.22	1.61±0.20	2.18±0.28*
Sult2a7	0.25±0.09	0.21±0.08	0.68±0.14	1.23±0.15*
Insrβ	1.32±0.24	1.45±0.32	1.18±0.19	1.02±0.10
Irs1	1.14±0.08	1.07±0.20	1.21±0.32	1.33±0.26
Pi3K	0.48±0.06	0.50±0.10	0.22±0.03	0.30±0.32
Akt1	0.83±0.21	0.95±0.15	0.88±0.42	0.98±0.21
mTor	0.38±0.26	2.92±0.49 $^{\phi}$	0.62±0.08	0.75±0.17
Pparγ	0.92±0.13	0.60±0.07*	0.32±0.35	0.39±0.34
Cd36	0.73±0.06	0.42±0.10*	0.64±0.08	0.67±0.09
Fasn	0.47±0.05	0.31±0.15	0.55±0.09	0.43±0.13
Gpat1	0.84±0.17	0.32±0.43	0.73±0.14	0.84±0.32
Gpat3	0.79±0.14	0.32±0.09*	0.61±0.14	0.66±0.17
Cidec	1.32±0.20	0.69±0.13*	1.02±0.29	1.14±0.36
Plin4	0.63±0.09	0.24±0.16*	0.59±0.25	0.48±0.10

	**male quad relative expression ratio**		**female quad relative expression ratio**	
	**WT**	***Akr1d1–/–***	**WT**	***Akr1d1–/–***
Insrβ	1.18±0.07	2.51±0.80	1.13±0.07	1.00±0.05
Irs1	0.65±0.04	1.34±0.21*	0.98±0.09	0.93±0.07
Pi3K	0.64±0.06	1.02±0.10	1.20±0.08	0.96±0.09$^{0.06}$
Akt1	1.15±0.08	1.33±0.26	1.14±0.07	0.98±0.06$^{0.09}$
mTor	0.43±0.10	0.30±0.32	0.37±0.08	0.53±0.13
Gsk3b	1.00±0.07	0.92±0.09	1.39±0.48	0.95±0.11
Pepck	0.39±0.09	0.56±0.13	0.43±0.13	0.50±0.16
Pyg1	0.32±0.03	0.47±0.03	0.44±0.08	0.95±0.17*
Cd36	0.55±0.05	0.98±0.21	0.49±0.04	0.37±0.04$^{0.06}$
Acc2	1.09±0.08	0.75±0.17	1.10±0.07	0.96±0.10
Cpt1	1.07±0.06	0.39±0.34	1.16±0.10	1.07±0.07

Acc1	0.80±0.21	0.67±0.09	0.42±0.07	0.39±0.06
Fasn	0.99±0.25	0.43±0.13	0.37±0.08	0.53±0.13
Dgat1	1.34±0.22	0.84±0.32	0.80±0.13	0.86±0.09
Gpam	1.15±0.07	0.66±0.17	1.00±0.10	0.87±0.09
Ppara	0.98±0.19	1.16±0.22	1.01±0.12	0.92±0.19
Pgc1a	0.85±0.09	0.81±0.07	0.93±0.16	0.89±0.11
Foxo1	1.16±0.07	1.18±0.06	1.11±0.20	1.05±0.19
Pdk4	0.56±0.08	0.67±0.11	0.64±0.14	0.57±0.14

	**male gonadal fat**		**female gonadal fat**	
	**relative expression ratio**		**relative expression ratio**	
	**WT**	***Akr1d1–/–***	**WT**	***Akr1d1–/–***
Pparg	0.73±0.13	0.86±0.16	0.63±0.21	0.59±0.12
Creb	0.98±0.15	1.17±0.06	1.07±0.13	0.99±0.10
Cd36	0.82±0.15	0.91±0.22	0.86±0.13	0.86±0.09
Atgl	0.69±0.30	0.98±0.12	0.71±0.15	0.82±0.15
Ucp1	0.98±0.19	0.95±0.13	1.13±0.22	1.22±0.07
Elovl3	0.74±0.10	0.79±0.09	0.68±0.12	0.75±0.20
Cpt2	1.21±0.13	1.32±0.10	1.22±0.12	0.99±0.30

Supplementary Table 3. Relative levels of serum lipids in mature (30-week) wildtype and *Akr1d1*$^{-/-}$ mice on control diet. Metabolon metabolomics relative signal intensity for serum monoacylglycerol, diacylglycerol and fatty acids. *p<0.05, **p<0.01, ØØp<0.0005, compared to wildtype within sex. Data are presented as mean ± se of n=10 mice.

	male serum		female serum	
	**WT**	***Akr1d1–/–***	**WT**	***Akr1d1–/–***
**monoacylglycerols**				
1-myristoylglycerol (14:0)	0.86±0.057	1.1±0.071	1.1±0.11	1.1±0.24
1-pentadecanoylglycerol (15:0)	1.1±0.099	1.2±0.081	1.0±0.095	1.2±0.26
1-palmitoylglycerol (16:0)	0.98±0.12	1.2±0.093	1.0±0.13	1.3±0.29
1-palmitoleoylglycerol (16:1)*	1.3±0.19	2.2±0.22*	0.63±0.069	0.59±0.098
1-oleoylglycerol (18:1)	1.0±0.16	1.4±0.13*	0.75±0.061	0.78±0.082
1-linoleoylglycerol (18:2)	1.0±0.092	1.6±0.19*	0.94±0.078	0.88±0.16
1-linolenoylglycerol (18:3)	0.99±0.098	1.4±0.14	0.98±0.11	0.97±0.23
1-dihomo-linolenylglycerol (20:3)	1.2±0.24	1.6±0.14*	0.76±0.071	0.75±0.10
1-arachidonylglycerol (20:4)	1.3±0.17	1.4±0.14	0.91±0.079	1.0±0.15
1-docosahexaenoylglycerol (22:6)	1.4±0.31	1.7±0.22	0.85±0.12	0.66±0.12
2-palmitoylglycerol (16:0)	1.0±0.12	1.4±0.34	1.1±0.11	1.2±0.25
2-palmitoleoylglycerol (16:1)*	1.3±0.18	2.0±0.20	0.61±0.068	0.57±0.092
2-oleoylglycerol (18:1)	1.1±0.23	1.5±0.14*	0.77±0.084	0.84±0.13
2-linoleoylglycerol (18:2)	1.0±0.083	1.5±0.19*	0.92±0.073	0.84±0.14
2-arachidonoylglycerol (20:4)	1.0±0.17	1.2±0.24	0.71±0.13	0.76±0.085
2-docosahexaenoylglycerol (22:6)*	1.4±0.33	1.4±0.27	0.8±0.16	0.76±0.085
1-dihomo-linoleoylglycerol (20:2)	1.2±0.13	1.7±0.19	0.81±0.077	0.84±0.11
**diacylglycerols**				
diacylglycerol (16:1/18:2 [2], 16:0/18:3 [1])	1.1±0.29	1.6±0.28	0.33±0.67	0.35±0.12
palmitoyl-oleoyl-glycerol (16:0/18:1) [2]	0.80±0.19	1.4±0.22*	0.28±0.033	0.35±0.092
palmitoyl-linoleoyl-glycerol (16:0/18:2) [1]	1.6±0.23	1.7±0.27	0.75±0.096	0.53±0.10
palmitoyl-linoleoyl-glycerol (16:0/18:2) [2]	1.1±0.22	1.7±0.32	0.62±0.096	0.56±0.18
palmitoleoyl-linoleoyl-glycerol (16:1/18:2)	1.4±0.19	2.9±0.31ϕϕ	0.59±0.064	0.49±0.071
palmitoyl-arachidonoyl-glycerol (16:0/20:4)	1.1±0.24	1.1±0.22	0.56±0.074	0.54±0.13
oleoyl-oleoyl-glycerol (18:1/18:1) [1]	0.79±0.16	1.8±0.32**	0.55±0.1	0.62±0.12
oleoyl-oleoyl-glycerol (18:1/18:1) [2]	1.2±0.22	2.2±0.37*	0.7±0.13	0.73±0.11
oleoyl-linoleoyl-glycerol (18:1/18:2) [1]	1.2±0.16	1.7±0.15**	0.78±0.066	0.6±0.087
oleoyl-linoleoyl-glycerol (18:1/18:2) [2]	1.0±0.14	1.9±0.16**	0.77±0.058	0.64±0.12
linoleoyl-linoleoyl-glycerol (18:2/18:2) [1]	1.2±0.20	1.7±0.12	0.72±0.10	0.64±0.13
linoleoyl-linolenoyl-glycerol (18:2/18:3) [1]	0.77±0.19	0.72±0.13	0.87±0.10	0.58±0.11
oleoyl-arachidonoyl-glycerol (18:1/20:4) [2]	1.1±0.20	1.4±0.21	0.58±0.087	0.82±0.15
linoleoyl-arachidonoyl-glycerol (18:2/20:4)	1.2±0.21	1.6±0.26	0.8±0.10	0.81±0.16
linoleoyl-arachidonoyl-glycerol (18:2/20:4)	1.0±0.15	1.6±0.21*	0.68±0.082	0.83±0.15
linoleoyl-docosahexaenoyl-glycerol	1.3±0.34	1.7±0.41	1.0±0.15	0.79±0.22

**polyunsaturated fatty acids**				
myristate (14:0)	1.0±0.095	1.4±0.12*	1.0±0.11	0.89±0.11
myristoleate (14:1n5)	1.1±0.22	1.5±0.21	0.89±0.15	0.83±0.14
pentadecanoate (15:0)	1.0±0.064	1.2±0.10	1.1±0.085	1.1±0.2
palmitate (16:0)	0.98±0.037	1.2±0.067*	0.99±0.073	0.85±0.11
palmitoleate (16:1n7)	1.1±0.10	1.7±0.17*	0.87±0.11	0.72±0.072
margarate (17:0)	0.99±0.037	1.1±0.067	1.1±0.099	0.96±0.14
10-heptadecenoate (17:1n7)	1.0±0.043	1.4±0.089*	1.1±0.13	0.81±0.059
stearate (18:0)	0.97±0.026	1.1±0.07	1.1±0.069	1.1±0.17
oleate/vaccenate (18:1)	0.95±0.076	1.3±0.095*	1.1±0.11	0.95±0.08
nonadecanoate (19:0)	1.2±0.065	0.96±0.099	0.82±0.078	0.64±0.094*
10-nonadecenoate (19:1n9)	1.0±0.047	1.4±0.11*	1.1±0.12	0.79±0.073*
arachidate (20:0)	1.1±0.063	1.5±0.099*	0.72±0.05	0.69±0.11
eicosenoate (20:1)	1.3±0.13	1.7±0.14*	0.79±0.075	0.64±0.12
behenate (22:0)	1.9±0.57	2.1±0.51	1.2±0.33	1.9±0.55
erucate (22:1n9)	1.6±0.22	2.4±0.22*	0.5±0.05	0.48±0.13
**polyunsaturated fatty acids**				
heneicosapentaenoate (21:5n3)	0.86±0.20	1.1±0.29	0.48±0.072	0.5±0.092
hexadecadienoate (16:2n6)	1.0±0.11	1.5±0.15*	0.94±0.11	0.81±0.059
stearidonate (18:4n3)	0.85±0.07	2.0±0.37*	1.0±0.092	0.84±0.068
eicosapentaenoate (EPA; 20:5n3)	0.98±0.096	1.4±0.15	1.1±0.096	0.83±0.087
docosapentaenoate (n3 DPA; 22:5n3)	1.2±0.14	1.7±0.23*	0.88±0.068	0.75±0.066
docosahexaenoate (DHA; 22:6n3)	0.96±0.13	1.1±0.12	1.1±0.073	0.88±0.10
docosatrienoate (22:3n3)	1.4±0.092	2.4±0.25 $^{\phi\phi}$	0.8±0.067	0.53±0.048**
nisinate (24:6n3)	1.1±0.11	1.8±0.26**	0.94±0.077	0.63±0.064*
linoleate (18:2n6)	0.98±0.065	1.3±0.095	1.1±0.14	0.92±0.072
linolenate [alpha or gamma; (18:3n3 or 6)]	1.0±0.13	1.3±0.098	1.2±0.15	0.96±0.11
dihomo-linolenate (20:3n3 or n6)	1.1±0.099	1.3±0.12	1.1±0.098	0.79±0.053*
arachidonate (20:4n6)	0.93±0.089	0.90±0.068	1.1±0.059	1.0±0.096
adrenate (22:4n6)	1.2±0.20	1.2±0.12	0.95±0.052	0.9±0.057
docosapentaenoate (n6 DPA; 22:5n6)	1.2±0.14	1.1±0.12	0.96±0.073	0.89±0.061
docosadienoate (22:2n6)	1.3±0.074	1.4±0.21 $^{\phi\phi}$	0.64±0.053	0.87±0.071
dihomo-linoleate (20:2n6)	1.1±0.075	1.4±0.11*	1.0±0.094	0.79±0.045*
mead acid (20:3n9)	1.1±0.19	1.0±0.12	1.1±0.11	1.1±0.076

Supplementary Table 4. Liver and serum bile acid concentrations in mature (30-week) wildtype and male *Akr1d1*$^{-/-}$ mice on high fat diet. Plasma bile acid concentration in liver and serum of wildtype and *Akr1d1*$^{-/-}$ mice. *p<0.05, **p<0.01, ***p<0.005, $^{\emptyset}$p<0.001, $^{\emptyset\emptyset}$p<0.0005, $^{\emptyset\emptyset\emptyset}$p<0.0001 compared to wildtype within sex. T, tauro; G, glycol; CA, cholic acid; CDCA, chenodeoxycholic acid; MCA, murocholic acid; DCA, deoxycholic acid; LCA, lithocholic acid; UDCA, ursodeoxycholic acid; HDCA, hyodeoxycholic acid; ND, not detected. Data are presented as mean ± se of n = 11-15 mice.

	**liver (pmol/mg)**		**serum (nM)**	
	**WT**	***Akr1d1–/–***	**WT**	***Akr1d1–***
**CA**	1.95±0.43	0.17±0.09**	578±147	43.5±16.9
**T-CA**	80.3±8.5	17.2±10.6***	115±16.5	ND
**G-CA**	0.34±0.06	0.09±0.06	ND	ND
**CDCA**	0.14±0.02	0.01±0.01***	36.4±8.3	16.1±0.4
**T-CDCA**	6.78±0.53	1.43±0.73$^{\phi\phi\phi}$	35.6±9.5	0.49±0.47
**G-CDCA**	ND	ND	10.2±1.4	8.06±0.20
**α-MCA**	0.53±0.9	0.08±0.05***	25.3±9.81	ND
**T-α-MCA**	17.1±1.35	4.17±1.90$^{\phi\phi}$	172±52.4	11.0±4.15
**β-MCA**	2.56±0.36	0.78±0.23**	76.0±28.0	12.5±5.7
**T-β-MCA**	38.3±3.9	13.8±4.3***	221±67	15.7±2.7
**ω-MCA**	0.56±0.10	0.13±0.03**	94.5±34.9	9.82±3.38
**T-ω-MCA**	30.0±3.4	9.18±2.24$^{\phi}$	497±103	62.4±11.7
**DCA**	0.21±0.05	ND**	404±95	21.0±5.9
**T-DCA**	9.06±1.31	0.97±0.38$^{\phi\phi}$	76.3±13.4	2.95±1.44
**LCA**	ND	ND	79.3±8.9	72.2±5.5
**T-LCA**	1.18±0.07	0.53±0.14***	1.00±0.18	ND
**G-LCA**	ND	ND	ND	ND
**UDCA**	0.15±0.04	0.07±0.04	59.5±14.9	16.7±1.7
**T-UDCA**	4.97±0.58	2.69±0.62	71.3±13.4	17.0±3.1
**G-UDCA**	ND	ND	5.92±0.22	5.53±0.22
**HDCA**	0.34±0.06	0.58±0.14	39.7±8.3	16.1±0.4
**7-oxo-DCA**	ND	ND	18.2±13.4	26.0±12.4
**T-7-oxo-LCA**	0.04±0.01	ND*	ND	ND
**12α-hydroxyl**	91.8±10.4	18.5±11.1$^{\phi\phi}$	1815±434	67.5±24.0
**non-12α-hydroxyl**	103±10	33.4±10.4$^{\phi\phi}$	1443±299	298±59
